# Nuclear membrane ruptures underlie the vascular pathology in a mouse model of Hutchinson-Gilford progeria syndrome

**DOI:** 10.1172/jci.insight.151515

**Published:** 2021-08-23

**Authors:** Paul H. Kim, Natalie Y. Chen, Patrick J. Heizer, Yiping Tu, Thomas A. Weston, Jared L.-C. Fong, Navjot Kaur Gill, Amy C. Rowat, Stephen G. Young, Loren G. Fong

**Affiliations:** 1 Department of Medicine,; 2 Department of Bioengineering,; 3 Department of Integrative Biology and Physiology, and; 4 Department of Human Genetics, UCLA, Los Angeles, California, USA.

**Keywords:** Vascular Biology, Cardiovascular disease, Genetic diseases, Mouse models

## Abstract

The mutant nuclear lamin protein (progerin) produced in Hutchinson-Gilford progeria syndrome (HGPS) results in loss of arterial smooth muscle cells (SMCs), but the mechanism has been unclear. We found that progerin induces repetitive nuclear membrane (NM) ruptures, DNA damage, and cell death in cultured SMCs. Reducing lamin B1 expression and exposing cells to mechanical stress — to mirror conditions in the aorta — triggered more frequent NM ruptures. Increasing lamin B1 protein levels had the opposite effect, reducing NM ruptures and improving cell survival. Remarkably, raising lamin B1 levels increased nuclear compliance in cells and was able to offset the increased nuclear stiffness caused by progerin. In mice, lamin B1 expression in aortic SMCs is normally very low, and in mice with a targeted HGPS mutation (*Lmna*^G609G^), levels of lamin B1 decrease further with age while progerin levels increase. Those observations suggest that NM ruptures might occur in aortic SMCs in vivo. Indeed, studies in *Lmna*^G609G^ mice identified NM ruptures in aortic SMCs, along with ultrastructural abnormalities in the cell nucleus that preceded SMC loss. Our studies identify NM ruptures in SMCs as likely causes of vascular pathology in HGPS.

## Graphical abstract

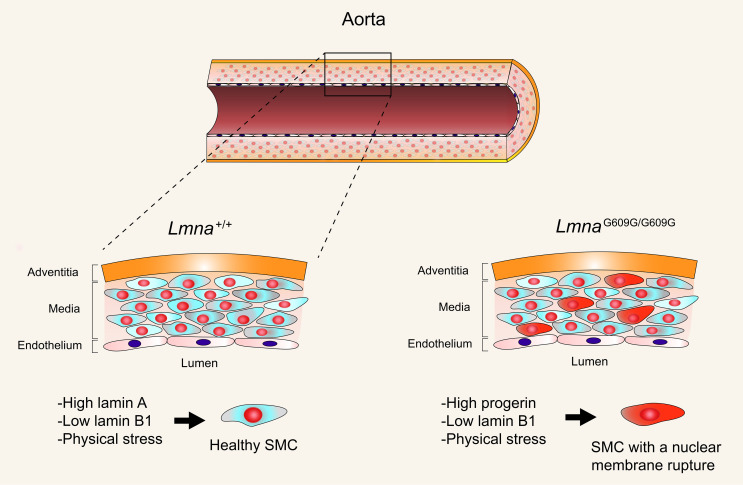

## Introduction

Hutchinson-Gilford progeria syndrome (HGPS) is a pediatric progeroid disorder caused by a point mutation in *LMNA* (the gene for prelamin A and lamin C) that causes aberrant prelamin A splicing and the production of an internally truncated prelamin A protein (progerin) (1, 2). Progerin cannot undergo the proteolytic processing step by ZMPSTE24 that normally converts farnesyl–prelamin A to mature lamin A; thus, progerin retains a farnesyl lipid anchor at its carboxyl terminus (3). In HGPS fibroblasts, progerin accumulates at the nuclear periphery and leads to a high frequency of misshapen nuclei (1, 4). Children with HGPS develop several aging-like phenotypes, but the most devastating is atherosclerosis in the coronary and cerebral arteries, which often leads to death by the mid-teenage years (5). The atherosclerosis in HGPS is unusual in that it occurs in the absence of common risk factors (e.g., hypercholesterolemia, smoking, and diabetes mellitus) (6). Histopathologic studies uncovered another atypical feature of the arterial disease in HGPS: the loss of medial smooth muscle cells (SMCs) (7, 8).

The loss of SMCs in large arteries, accompanied by thickening of the adventitia, is also observed in transgenic and gene-targeted mouse models of HGPS (9–11). The severity of the arterial disease depends on levels of progerin expression; the arterial pathology is more severe — and the onset is earlier — in mice that have 2 targeted HGPS mutant alleles as compared with only 1 (12, 13). The arterial disease is less severe in HGPS mice treated with a farnesyltransferase inhibitor (14), an antisense oligonucleotide that alters RNA splicing and reduces progerin expression (11), or by inhibiting N-acetyltransferase 10 (15), endoplasmic reticulum stress (16), MMP13 (17), or ICMT (18).

Mechanical stress is relevant to the vascular pathology in HGPS. In cultured SMCs that express progerin, mechanical stretching increases DNA damage and cell death (12). Disrupting the linker of the nucleoskeleton and cytoskeleton (LINC) complex, which reduces force transmission to the nucleus (19, 20), reduces DNA damage and increases cell survival. Also, in gene-targeted mice expressing progerin (*Lmna*^G609G^–knock-in mice), the loss of medial SMCs is most pronounced in segments of the aorta that are subjected to high hemodynamic stress (e.g., inner curvature of the ascending thoracic aorta) (12, 21). Of note, disrupting the LINC complex in the SMCs of *Lmna*^G609G^ mice markedly reduces arterial pathology (12).

In *Lmna*^G609G^ mice, nuclear abnormalities and cell death are widespread in medial SMCs but absent in intimal endothelial cells (12), even though both cell types are subjected to pulsatile hemodynamic forces. One potential clue to this discrepancy relates to differences in the composition of the nuclear lamina. In both WT and *Lmna*^G609G^ mice, lamin B1 expression is very low in medial SMCs but is robust in endothelial cells (12). Lamin B1, like progerin, is tethered to the inner nuclear membrane (NM) by a farnesyl lipid anchor (22), but whether the physiologically low levels of lamin B1 in medial SMCs render cells more susceptible to progerin toxicity is unknown. Knocking down lamin B1 expression in cultured osteosarcoma cells was reported to trigger NM ruptures (23), but the relevance of that finding to aortic SMCs in vivo is open to question because plating cells on plastic dishes increases susceptibility to NM ruptures (24, 25). NM ruptures have been observed in cortical neurons of lamin B1–deficient mouse embryos (26), but the relevance of that finding to arterial SMCs is also questionable because embryonic neurons, unlike arterial SMCs, do not express lamin A or lamin C (27, 28), 2 nuclear lamins that are crucial for the integrity of the nuclear envelope.

We believe that the low levels of lamin B1 in arterial SMCs are normally well tolerated because SMCs produce high levels of lamin A and lamin C (12, 29). However, we suspected that the low levels of lamin B1, combined with progerin expression, could be relevant to the vascular pathology of HGPS. Specifically, we hypothesized that progerin expression in SMCs, particularly when the cells are subjected to mechanical stress, could reduce NM integrity and trigger NM ruptures, resulting in reduced cell survival. We also hypothesized that increasing lamin B1 expression in progerin-expressing SMCs (so as to model the situation in intimal endothelial cells) would render SMCs less susceptible to NM ruptures. In addressing our hypotheses, we were keenly aware that cell culture studies are an imperfect model for arterial SMCs and HGPS pathology. Therefore, we also examined, in *Lmna*^G609G^ mice, whether progerin triggers NM ruptures in arterial SMCs but not in endothelial cells, and whether the onset of NM ruptures in SMCs correspond temporally to the emergence of the hallmark arterial pathology of HGPS.

## Results

### Progerin expression causes NM ruptures in cultured SMCs.

**Figure 1:**
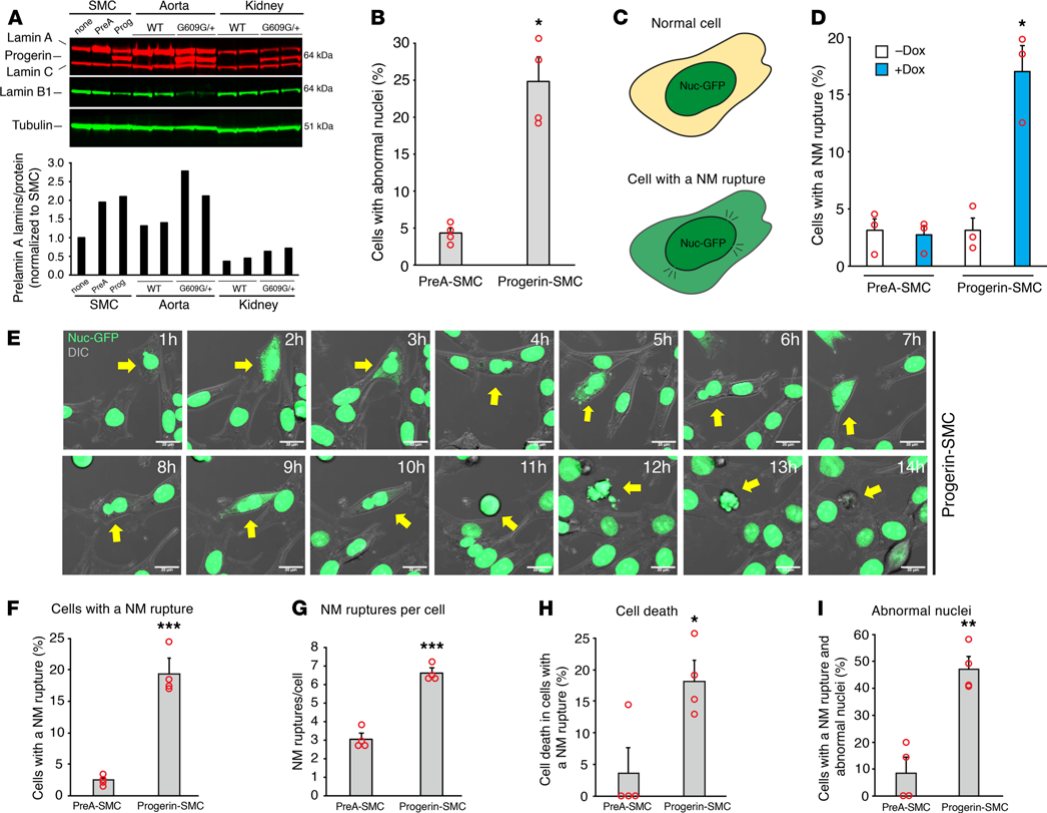
Progerin expression causes NM ruptures in cultured SMCs. (**A**) Western blot analysis of SMCs expressing prelamin A (PreA) and progerin (Prog), and in the aorta and kidney of WT and *Lmna*^G609G/+^ mice. The bar graph shows the expression of lamin A plus progerin, relative to total protein (Supplemental Figure 1C). (**B**) Bar graph showing that progerin increases the frequency of abnormally shaped nuclei in SMCs, as judged by live-cell microscopy (mean ± SEM, *n* = 4 experiments; Student’s *t* test, *P < 0.001). (**C**) Illustration depicting a NM rupture, defined as the escape of nuclear-targeted GFP (Nuc-GFP; green) into the cytoplasm (tan). (**D**) Bar graph showing that progerin causes NM ruptures in SMCs (mean ± SEM, *n* = 3 experiments; Student’s *t* test, *P < 0.01). NM ruptures were measured in fixed cells 48 hours after adding Dox (blue). (**E**) Fluorescence microscopy images of live SMCs expressing progerin and Nuc-GFP (green) at 1-hour intervals. A DIC image (grey) is superimposed. The yellow arrow points to a cell followed for 14 hours by time-lapse microscopy (see Supplemental Video 1; images were captured every 10 minutes). The cell had several NM ruptures and eventually died. Scale bar: 20 μm. (**F**–**I**) Characteristics of NM ruptures in PreA-SMC and Progerin-SMC derived from time-lapse microscopy studies. Bar graphs show the percentage of cells with a NM rupture; the number of NM ruptures per cell; the percentage of cells with a NM rupture that die; and the percentage of cells with a NM rupture and abnormal-shaped nuclei (mean ± SEM, *n* = 4 experiments; Student’s *t* test, *P < 0.02, **P < 0.002, ***P < 0.001). The results from the individual experiments are shown in Supplemental Figure 1E.

We generated aortic SMCs expressing nuclear-targeted GFP (Nuc-GFP) and doxycycline-inducible (Dox-inducible) constructs for WT prelamin A (PreA-SMC) or progerin (Prog-SMC). Dox levels were adjusted to achieve lamin A (or progerin) levels similar to those in aortas of WT mice (*Lmna*^+/+^) or mice with a targeted HGPS mutation (*Lmna*^G609G/+^) (Figure 1A). Both lamin A and progerin were located in the cell nucleus (Supplemental Figure 1A; supplemental material available online with this article; https://doi.org/10.1172/jci.insight.151515DS1). After 2 days, progerin increased the frequency of misshapen nuclei in both live SMCs (Figure 1B) and fixed cells (Supplemental Figure 1B). NM ruptures, evident from the escape of a nuclear-targeted fluorescent protein into the cytoplasm (23, 30, 31) (Figure 1C), were detected by fluorescence microscopy. In the absence of Dox, the frequency of NM ruptures was low in PreA-SMCs and Prog-SMCs (~3%) (Figure 1D). In the presence of Dox, 17% of Prog-SMCs exhibited a NM rupture, whereas only approximately 3% of PreA-SMCs had a rupture. In Prog-SMCs, the frequency of ruptures increased with higher levels of progerin expression (Supplemental Figure 1D).

To define the dynamics of NM ruptures, Prog-SMCs were monitored by time-lapse microscopy (images were taken every 10 minutes for 24 hours). Snapshots of Prog-SMCs at 1-hour intervals are shown in Figure 1E (also see Supplemental Video 1). Our analysis of time-lapse experiments (915 PreA-SMCs and 735 Prog-SMCs) revealed that NM ruptures were more frequent in Prog-SMCs than in PreA-SMCs (18% vs. 2.5%; *P* < 0.001) (Figure 1F) and often occurred repetitively (Figure 1G) (see Supplemental Videos 2 and 3). Cell death also occurred more frequently in Prog-SMCs with NM ruptures (25/141 cells vs. only 1/21 in PreA-SMCs; *P* < 0.02) (Figure 1H). NM ruptures were more frequent in Prog-SMCs that had a misshapen nucleus (Figure 1I). The results from individual experiments are shown in Supplemental Figure 1E.

### Impact of lamin B1, mechanical stress, and progerin farnesylation on NM ruptures.

**Figure 2:**
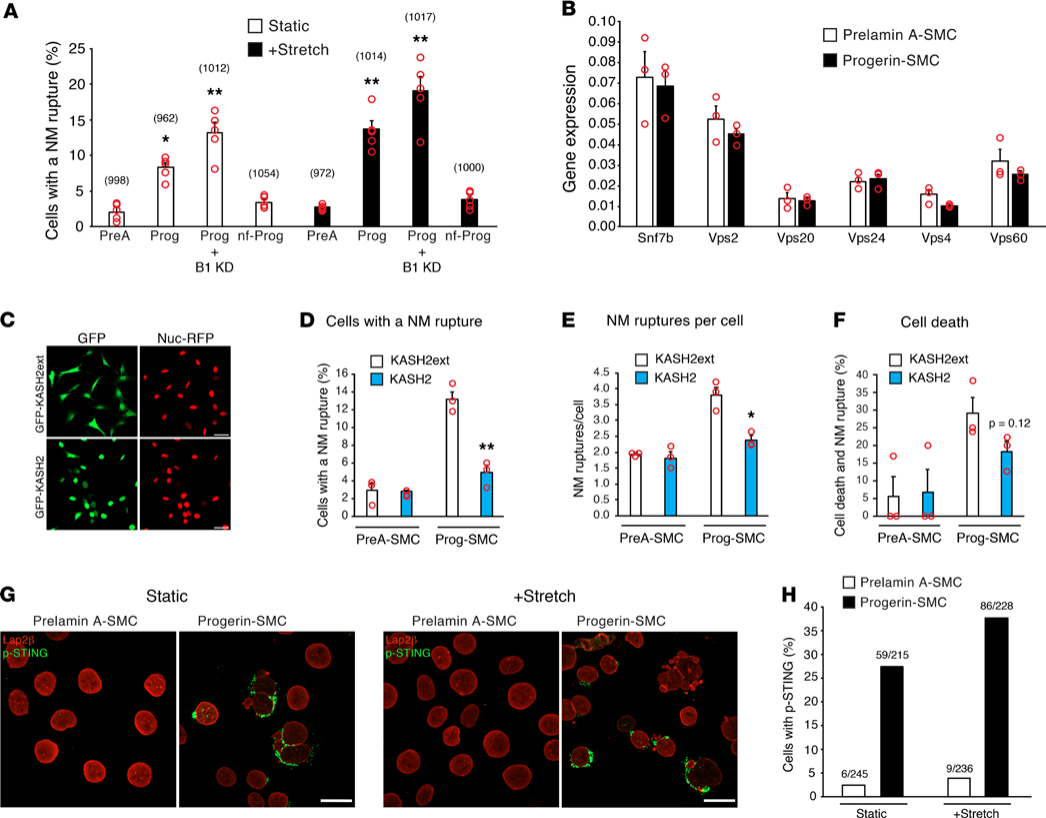
Impact of lamin B1, mechanical stress, and progerin farnesylation on NM ruptures. (**A**) Bar graph comparing the effects of prelamin A (PreA), progerin (Prog), lamin B1 knockdown (B1 KD), and nf-Prog on NM ruptures in static (white bars) and stretched (black bars) SMCs (mean ± SEM, *n* = 5 experiments). Differences were compared with static SMCs expressing prelamin A by 2-way ANOVA (*P < 0.005, **P < 0.0001). The total numbers of cells examined are shown in parentheses above each bar. (**B**) Bar graph showing transcript levels for genes involved in NM repair (mean ± SEM, *n* = 3 experiments; Student’s *t* test, all nonsignificant). (**C**) Confocal fluorescence microscopy images showing SMCs expressing Nuc-RFP (red) and either GFP-KASH2ext (green) or GFP-KASH2 (green). Scale bar: 20 μm. (**D**–**F**) Characterization of NM ruptures in live PreA-SMCs and Prog-SMCs expressing KASH2ext (white bars) or KASH2 (blue bars). Bar graphs show the percentage of cells with a NM rupture; the number of NM ruptures per cell; and the percentage of cells with a NM rupture that die (mean ± SEM, *n* = 3 experiments; Student’s *t* test, *P < 0.01, **P < 0.005). (**G**) Confocal fluorescence microscopy images showing increased phosphorylated-STING (p-STING; green) in Progerin-SMCs. SMCs were examined in both static (left) and stretched (right) conditions. PreA-SMCs were included as a control. Lap2β staining is shown in red. Scale bar: 20 μm. (**H**) Bar graph showing the percentage of cells staining positive for p-STING in PreA-SMCs (white bars) and Prog-SMCs (black bars) in static and stretched conditions in a representative experiment. The ratios above each bar show the number of cells positive for p-STING over the total number of cells assessed.

To examine the effects of low lamin B1 expression and mechanical stress on NM ruptures — so as to model conditions in the aorta — Prog-SMCs and PreA-SMCs were treated with a *Lmnb1* siRNA (or a control siRNA), plated on collagen-coated polydimethylsiloxane (PDMS) membranes, and subjected to either uniaxial stretching (2 mm, 0.5 Hz for 2 hours) or static conditions. Western blots documented reduced lamin B1 levels in *Lmnb1* siRNA–treated cells (Supplemental Figure 2A). The lower levels of lamin B1 expression increased NM ruptures in Prog-SMCs by 58% in static conditions (*P* < 0.05) and 40% in stretch conditions (*P* < 0.02) (Figure 2A) but had no effect on ruptures in PreA-SMCs (Supplemental Figure 2B). Stretching increased NM ruptures in both untreated Prog-SMCs (1.6-fold vs. static Prog-SMCs; *P* < 0.02) and *Lmnb1* siRNA–treated Prog-SMCs (2.3-fold vs. static Prog-SMCs; *P* < 0.0001). The expression of a nonfarnesylated (nf) progerin (nf-Prog), a progerin that terminated with –SSIM rather than –CSIM, a modification to the C-terminal CaaX signal sequence that blocks progerin farnesylation) (Supplemental Figure 2A), did not trigger NM ruptures in either static or stretched SMCs (Figure 2A).

The endosomal sorting complex required for transport III (ESCRT-III) protein complex plays a role in repairing NM ruptures (31, 32). Transcript levels for components of the ESCRT-III protein complex (*Vps20*, *Snf7b*, *Vps24*, *Vps2*, *Vps4*, and *Vps60*) were not different in Prog-SMCs and PreA-SMCs (Figure 2B).

Finding increased NM ruptures in stretched Prog-SMCs implied that mechanical strain contributes to ruptures. To explore that idea, we expressed a nuclear-targeted red fluorescence protein (RFP) (Nuc-RFP) in SMCs and then disrupted the LINC complex with a GFP-tagged Klarsicht/Anc-1, Syne homology (KASH) domain of nesprin-2 (GFP-KASH2) (33) (Figure 2C). NM ruptures, documented by the escape of Nuc-RFP into the cytoplasm, were reduced by KASH2 expression in Prog-SMCs (compared with KASHext, an inactive KASH2 protein) (Figure 2, D and E). There was a trend of reduced cell death in KASH2-expressing Prog-SMCs but the differences did not achieve statistical significance (*P* = 0.12) (Figure 2F). KASH2 expression had no effect on NM ruptures in PreA-SMCs (Figure 2, D and E). The results from individual experiments are shown in Supplemental Figure 2C.

NM ruptures result in the intermixing of nuclear and cytoplasmic contents (23, 30, 34). To determine if NM ruptures activate the cGAS-STING pathway (32, 35), we assessed phosphorylated-STING by immunofluorescence microscopy. Phosphorylated-STING was detected at a higher frequency in both static and stretched Prog-SMCs (Figure 2, G and H).

### Lamin B1 reduces progerin’s toxicity and association with NMs.

**Figure 3:**
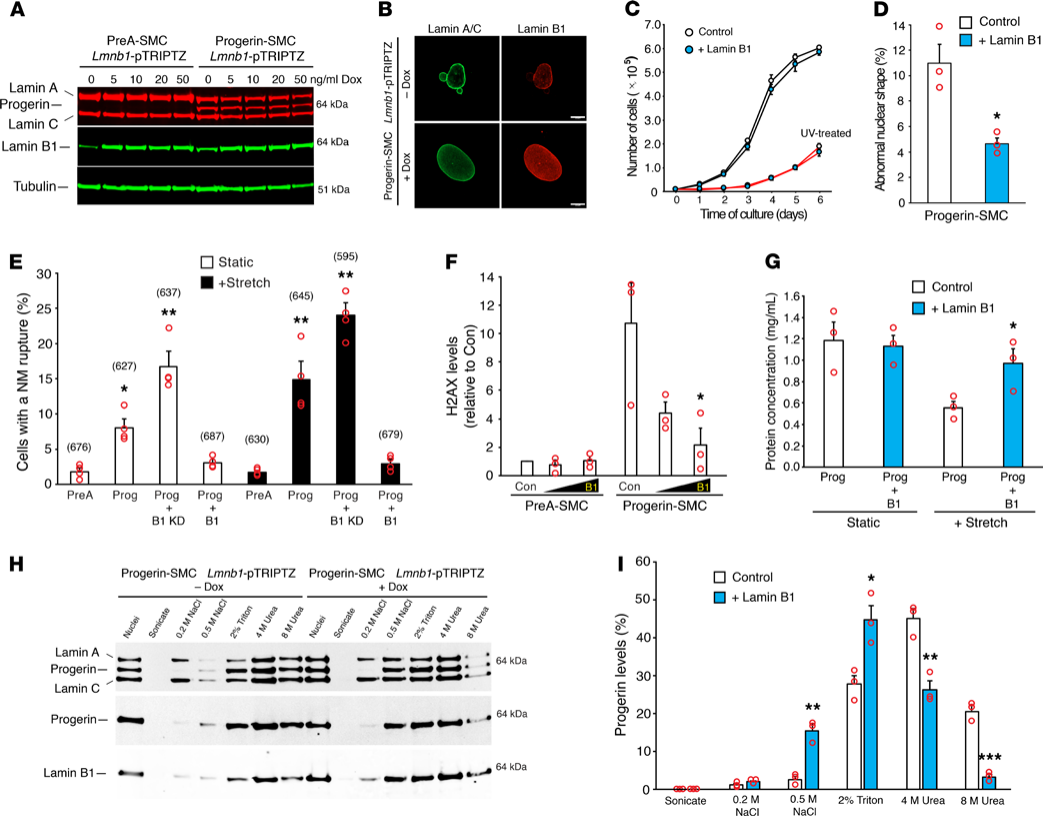
Lamin B1 reduces progerin’s toxicity and association with NMs. (**A**) Western blot showing Dox-induced expression of mouse lamin B1 in PreA-SMCs and Prog-SMCs. (**B**) Microscopy images showing lamin B1 expression (red) in Prog-SMCs. Scale bar: 10 μm. (**C**) Growth curves showing that induced lamin B1 expression (blue circles) does not affect cell growth in control (black lines) or UV-treated SMCs (red lines). Mean ± SEM (*n* = 3 experiments). (**D**) Bar graph showing that lamin B1 overexpression (blue) reduces abnormal nuclear shape in Prog-SMCs (mean ± SEM, *n* = 3 experiments; Student’s *t* test, *P < 0.02). (**E**) Bar graph comparing the effects of lamin B1 knockdown (B1 KD) and lamin B1 overexpression (B1) on NM ruptures in Prog-SMCs under static (white bars) and stretched (black bars) conditions (mean ± SEM, *n* = 4 experiments). NM ruptures were compared with static PreA-SMCs by 2-way ANOVA (*P < 0.05, **P < 0.0001). The total numbers of cells examined are shown in parentheses above each bar. (**F**) Bar graph showing that lamin B1 reduces H2AX-γ levels in Prog-SMCs (mean ± SEM, *n* = 3 experiments). Levels were compared with control (Con) Prog-SMCs by ANOVA (*P < 0.01). (**G**) Bar graph showing that lamin B1 reduces cell death in stretched Prog-SMCs (mean ± SEM, *n* = 3 experiments; Student’s *t* test, *P < 0.05). Dead cells detach from membranes, reducing cell protein on the membranes (12). (**H**) Western blot showing that lamin B1 expression increases the solubility of progerin. Nuclei from Prog-SMCs were sequentially extracted as described in Methods and the soluble extracts analyzed by western blotting. (**I**) Bar graph comparing the extraction profiles for progerin in Prog-SMCs (white) and Prog-SMCs plus lamin B1 (blue) (mean ± SEM, *n* = 3 experiments; Student’s *t* test, *P < 0.02, **P < 0.01, ***P < 0.001).

To determine if increasing lamin B1 levels alter the frequency of progerin-induced NM ruptures, we created Prog-SMCs harboring a Dox-inducible lamin B1 construct. Inducing lamin B1 (Figure 3, A and B) did not alter the growth of Prog-SMCs (Figure 3C) but reduced the number of cells with misshapen nuclei (Figure 3D). Induced lamin B1 expression also markedly reduced NM ruptures in Prog-SMCs under both static (61% decrease) and stretched (80% decrease) conditions (Figure 3E). Also, lamin B1 expression resulted in less DNA damage in Prog-SMCs (but not in PreA-SMCs), as judged by H2AXγ levels (Figure 3F). Finally, lamin B1 increased cell survival in stretched Prog-SMCs (Figure 3G).

The farnesyl lipid anchor is thought to tether lamin B1 and progerin to the inner NM (36–38). To determine if increased lamin B1 interferes with progerin’s association with the nuclear envelope, Prog-SMC nuclei were sequentially extracted with NaCl, Triton, and urea (39). In the absence of lamin B1 induction (–Dox), approximately 30% of the progerin appeared in the NaCl/Triton fractions, whereas approximately 70% appeared in the urea fractions. With lamin B1 expression (+Dox), 60% of the progerin appeared in the NaCl/Triton fractions, indicating reduced association of progerin with the nuclear envelope (Figure 3, H and I). In control studies, we found, as expected, a high percentage of nf-Prog (~60%) in the NaCl/Triton fractions (Supplemental Figure 3, A and B).

### Lamin B1 decreases nuclear stiffness.

**Figure 4:**
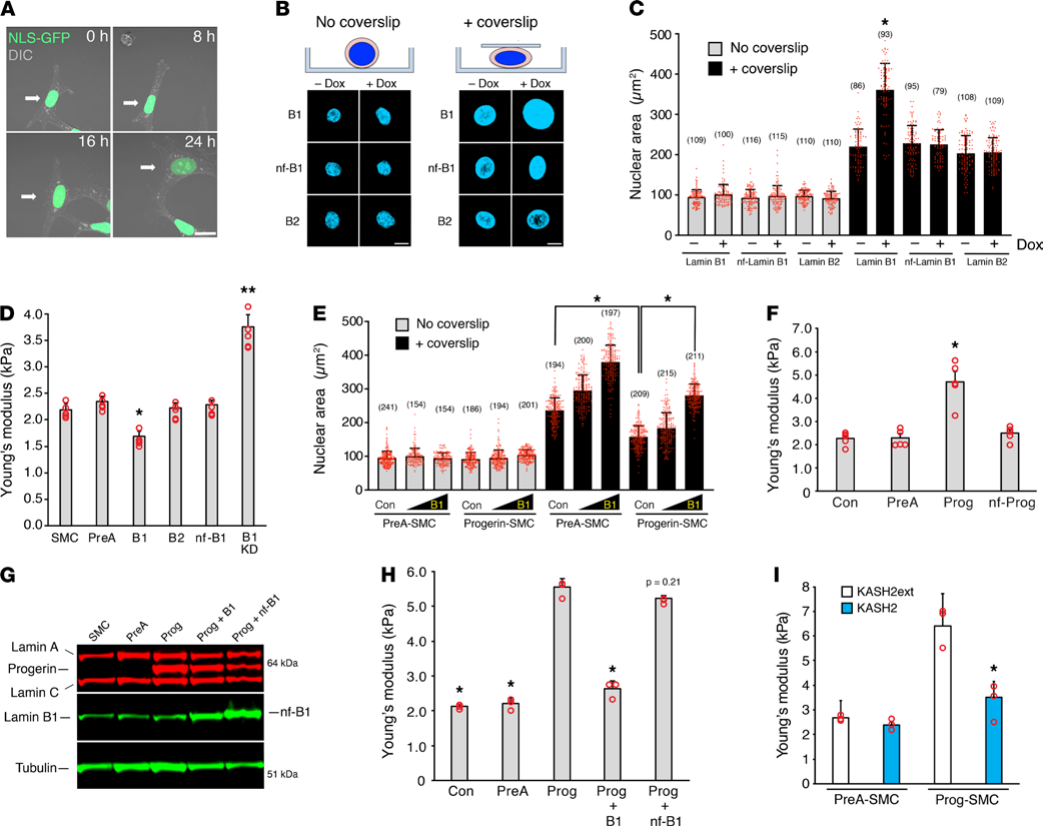
Lamin B1 decreases nuclear stiffness. (**A**) Microscopy images of a live SMC (arrow) expressing Nuc-GFP (green) and increased amounts of lamin B1 (see Supplemental Video 4). Scale bar: 20 μm. (**B**) Microscopy images of Hoechst-stained nuclei in suspended cells expressing lamin B1 (B1), nf-lamin B1 (nf-B1), or lamin B2 (B2) before (left) and after (right) compressing the cells with a glass coverslip. Scale bar: 10 μm. Nuclear lamin expression was induced with Dox (see Supplemental Figure 4A). (**C**) Inducing lamin B1 expression (+Dox) increases nuclear area (mean ± SD, numbers of cells examined are reported above each bar; Student’s *t* test, *P < 0.001). (**D**) Lamin B1 (B1) decreases nuclear stiffness (Young’s modulus) in SMCs, as measured by AFM (mean ± SEM, *n* = 5 experiments). Measurements were compared with noninduced SMCs by ANOVA (*P < 0.002, **P < 0.0001). (**E**) Lamin B1 increases nuclear size (spreading) in PreA-SMCs and Progerin-SMCs. Nuclei size was measured as in **C** (mean ± SD; Student’s *t* test, *P < 0.001). (**F**) Progerin (Prog) but not nf-Prog increases nuclear stiffness in SMCs (mean ± SEM, *n* = 5 experiments). Expression data are shown in Supplemental Figure 4F. All measurements were compared with control SMCs (Con) by ANOVA (*P < 0.0001). (**G**) Western blot showing increased expression of lamin B1 (B1) and nf-B1 in SMCs examined in **H**. (**H**) Increasing lamin B1 expression but not nf-B1 reduces nuclear stiffness in Prog-SMCs (mean ± SEM, *n* = 3 experiments). Measurements were compared with SMCs expressing progerin by ANOVA (*P < 0.0001). (**I**) Disrupting the LINC complex with KASH2 (blue bars) reduces nuclear stiffness in Prog-SMCs (mean ± SEM; *n* = 3 experiments). Nuclear stiffness was compared with cells expressing the inactive mutant KASH2ext (white bars) by the Student’s *t* test (*P < 0.01).

In live-cell imaging studies, we noted that SMC nuclei appeared larger in cells expressing lamin B1 (Figure 4A and Supplemental Video 4). Since nuclear size is determined by chromosomal content, nuclear lamina stiffness, and cytoskeletal elements (i.e., microfilaments and microtubules), we hypothesized that lamin B1 increased nuclear size by decreasing nuclear stiffness. To pursue this, we expressed prelamin A, lamin B1, nf-lamin B1, and lamin B2 in SMCs (Supplemental Figure 4A) and examined the nuclear size in suspension cultures stained with Hoechst at baseline and after compressing the cells with a glass coverslip (Figure 4B). The purpose of the coverslip was to mimic the pressure on the nucleus by cytoskeletal elements and to apply a uniform level of force. The coverslip increased nuclear area in all SMCs, as expected, but the increase was particularly striking in the lamin B1–transduced SMCs (Figure 4C). Increased nuclear area was also observed in fibroblasts transduced with lamin B1 (Supplemental Figure 4, B and C).

We suspected that lamin B1 expression increased nuclear size by decreasing nuclear stiffness. By atomic force microscopy (AFM) measurements over the cell nucleus, lamin B1 expression reduced nuclear stiffness by 23% (*P* < 0.002), whereas a lamin B1 knockdown increased nuclear stiffness by approximately 70% (*P* < 0.0001). Increased expression of prelamin A, lamin B2, and nf-lamin B1 had no effect on nuclear stiffness (Figure 4D and Supplemental Figure 4D). The increase in nuclear area in SMCs expressing lamin B1 (induced with 10 ng/mL Dox) was not accompanied by increased expression of nuclear envelope proteins (Supplemental Figure 4E), suggesting that lamin B1 did not increase nuclear size by inducing larger nuclei.

Lamin B1 expression also increased nuclear area in cells expressing progerin that were compressed with a glass coverslip (Figure 4E). However, the extent of nuclear spreading was significantly lower in Prog-SMCs than in PreA-SMCs, implying that progerin expression made nuclei stiffer. Indeed, by AFM, progerin increased nuclear stiffness more than 2-fold (*P* < 0.0001), whereas prelamin A and nf-Prog had no effect (Figure 4F and Supplemental Figure 4F). The increased nuclear stiffness in Prog-SMCs was reduced by expression of lamin B1 and KASH2, but not by nf-lamin B1 or KASH2ext (Figure 4, G–I).

### Progerin levels increase with age in Lmna^G609G/+^ mice whereas lamin B1 levels decrease.

**Figure 5:**
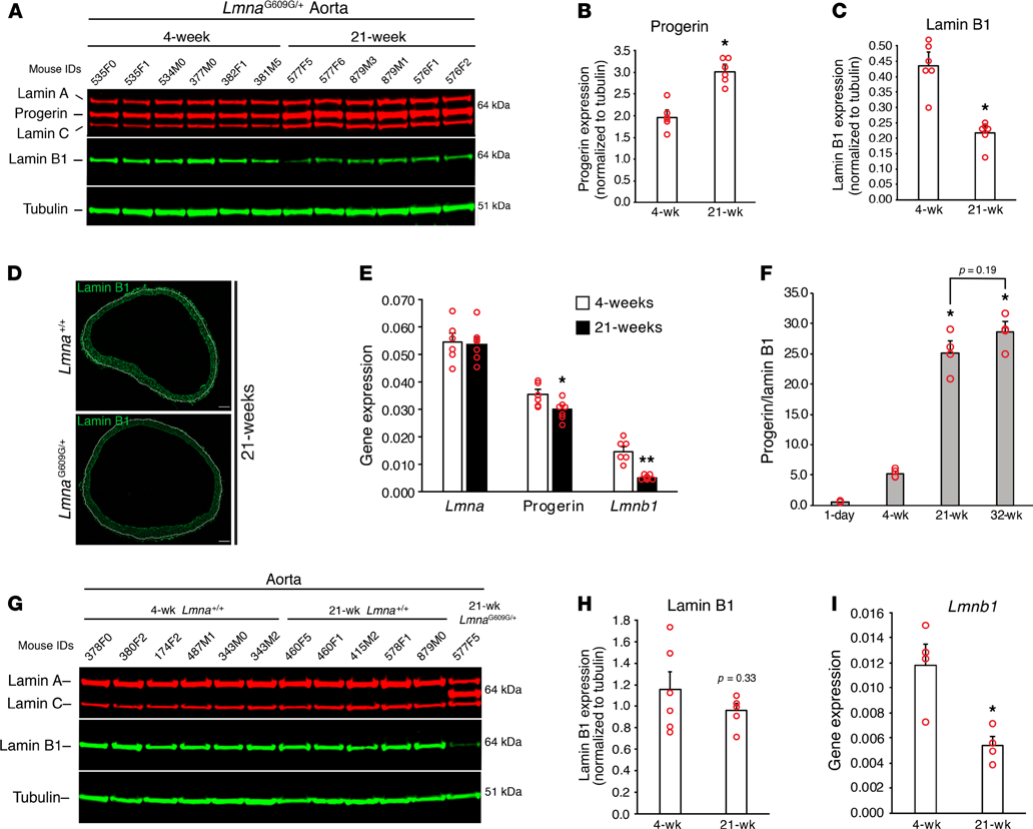
Progerin levels increase with age in *Lmna*^G609G/+^ mice whereas lamin B1 levels decrease. (**A**) Western blot comparing the expression of progerin and lamin B1 in the aorta of 4- and 21-week-old *Lmna*^G609G/+^ mice. Mouse IDs are shown above each sample. (**B** and **C**) Bar graphs showing progerin and lamin B1 levels, relative to tubulin, for the western blot in (**A**) (mean ± SEM, *n* = 6 mice/group; Student’s *t* test, *P < 0.001). (**D**) Immunofluorescence microscopy images of aortic rings showing lamin B1 (green) expression is reduced in SMCs in 21-week-old *Lmna*^G609G/+^ mice. The border between the media and adventitia is marked with a white line. Scale bar: 100 μm. (**E**) Quantitative RT-PCR studies showing *Lmna*, progerin, and *Lmnb1* transcript levels in aortas from 4-week-old (*n* = 6 mice) and 21-week-old (*n* = 7 mice) *Lmna*^G609G/+^ mice (mean ± SEM; Student’s *t* test, *P < 0.05, **P < 0.001. (**F**) Bar graph comparing the progerin-to-lamin B1 ratio in the aorta from 1-day-old *Lmna*^G609G/+^ mice (*n* = 2), and from 4-, 21-, and 32-week-old *Lmna*^G609G/+^ mice (*n* = 4) for the western blot in Supplemental Figure 5D. Comparisons were made to 4-week-old *Lmna*^G609G/+^ mice by ANOVA (*P < 0.0001). (**G**) Western blot comparing the expression of lamin B1 in the aorta from 4- (*n* = 6) and 21- (*n* = 5) week-old *Lmna*^+/+^ mice. Mouse IDs are shown above each sample. For comparison, a sample from a 21-week-old *Lmna*^G609G/+^ mouse is included. (**H**) Bar graph showing the expression of lamin B1, relative to tubulin, in the western blot in **G** (mean ± SEM; Student’s *t* test). (**I**) Bar graph showing *Lmnb1* expression in the aorta of 4- and 21-week-old *Lmna*^+/+^ mice (mean ± SEM, *n* = 4 mice/group; Student’s *t* test, *P < 0.02).

NM rupture frequency in cultured SMCs depends on the expression levels of progerin and lamin B1. To explore the relevance of these findings in living mice, we began by using western blots to compare progerin and lamin B1 expression in aortas of 4- and 21-week-old *Lmna*^G609G/+^ mice (Figure 5A). Progerin levels in the aorta of *Lmna*^G609G/+^ mice were approximately 50% higher at 21 weeks than at 4 weeks (Figure 5B), while lamin B1 levels were approximately 50% lower (Figure 5C and Supplemental Figure 5, A–C) (both *P* < 0.001). Microscopy revealed that the lower levels of lamin B1 were due to reduced expression in medial SMCs (Figure 5D). The age-dependent change in lamin B1 protein levels was accompanied by a 67% decrease in *Lmnb1* expression; however, the increase in progerin protein levels could not be explained by changes in gene expression (Figure 5E). At 32 weeks, the levels of progerin protein, relative to lamin B1, stabilized (Figure 5F and Supplemental Figure 5, D and E).

The decrease in lamin B1 levels in the aorta from 4–21 weeks was observed only in *Lmna*^G609G/+^ mice. In WT mice, lamin B1 levels were not significantly reduced at 21 weeks (Figure 5, G and H) (*P* = 0.33), despite the fact that *Lmnb1* transcript levels had fallen by more than 50% (Figure 5I).

### NM ruptures and ultrastructural abnormalities in aortic SMCs precede SMC loss in Lmna^G609G/G609G^ mice.

**Figure 6:**
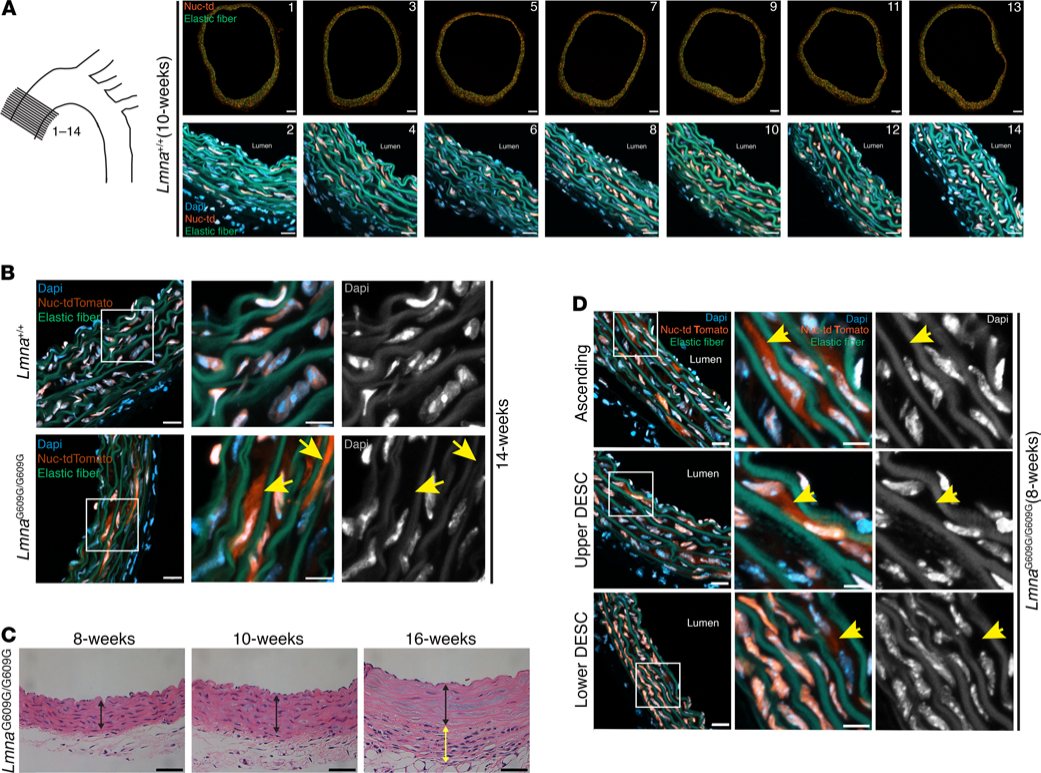
NM ruptures in aortic SMCs precede SMC loss in *Lmna*^G609G/G609G^ mice. (**A**) Expression of Nuc-tdTomato in SMCs is uniform in the ascending aorta. Fourteen sequential cross sections through the ascending aorta were collected every 100 μm from a *Lmna*^+/+^ mouse. Half of the sections (odd-numbered sections; top row) were imaged at low magnification to visualize the entire cross section. Scale bar: 100 μm. The even-numbered sections (bottom row) were imaged at higher magnification to visualize Nuc-tdTomato (orange) in nuclei stained with DAPI (blue). Scale bar: 20 μm. (**B**) Confocal fluorescence microscopy images of the ascending aorta from a 14-week-old *Lmna*^+/+^ and *Lmna*^G609G/G609G^ mouse. The boxed regions are shown at higher magnification in the middle and far-right columns. The colored images show DAPI (blue), elastic fibers (green), and Nuc-tdTomato (orange). The yellow arrows (middle column) point to Nuc-tdTomato outside of an SMC nucleus. To help visualize the boundaries of nuclei, the DAPI stain (white) is shown by itself in the far-right column. Scale bars: 20 μm (left column); 10 μm (middle column). (**C**) H&E-stained sections of the inner ascending aorta from 8-, 10-, and 16-week-old *Lmna*^G609G/G609G^ mice. The black line spans the medial layer and the yellow line spans the thickened and fibrotic adventitia in the 16-week-old mouse. Scale bar: 20 μm. Note the loss of SMC nuclei in the aorta from the 16-week-old mouse. (**D**) Confocal fluorescence microscopy images of the ascending, upper descending, and lower descending thoracic aorta from an 8-week-old *Lmna*^G609G/G609G^ mouse. Boxed regions are analyzed as described in (**B**). Scale bars: 20 μm (left column); 10 μm (middle column).

Finding high progerin levels but low lamin B1 levels in aortas of *Lmna*^G609G/+^ mice suggested that we might find NM ruptures in vivo in aortic SMCs. To explore this possibility, we bred WT and *Lmna*^G609G/G609G^ mice with a nuclear-targeted tdTomato (Nuc-tdTomato) transgene (26). By fluorescence microscopy, Nuc-tdTomato expression was uniform in the thoracic aorta and was confined to the cell nucleus (Figure 6A).

We used fluorescence microscopy to visualize Nuc-tdTomato in aortas of *Lmna*^G609G/G609G^ mice at 14 weeks of age. At this time point, loss of medial SMCs is clearly evident (12). We found frequent NM ruptures in medial SMCs of *Lmna*^G609G/G609G^ mice, as judged by the escape of Nuc-tdTomato into the cytoplasm (Figure 6B). No NM ruptures were observed in aortic endothelial cells consistent with the absence of endothelial cell loss in *Lmna*^G609G/G609G^ mice. In WT mice, Nuc-tdTomato was confined to the nucleus of SMCs (Figure 6B).

We next asked whether the appearance of NM ruptures in the aortic SMCs of *Lmna*^G609G/G609G^ mice precedes the onset of SMC loss. Loss of SMCs in *Lmna*^G609G/G609G^ mice was detectable at 10 weeks of age but absent at 8 weeks (Figure 6C). However, at 8 weeks of age, we observed frequent NM ruptures in medial SMCs of *Lmna*^G609G/G609G^ mice in the ascending aorta and upper descending aorta and less frequent ruptures in the lower descending aorta (Figure 6D and Supplemental Figure 6, A and B).

**Figure 7:**
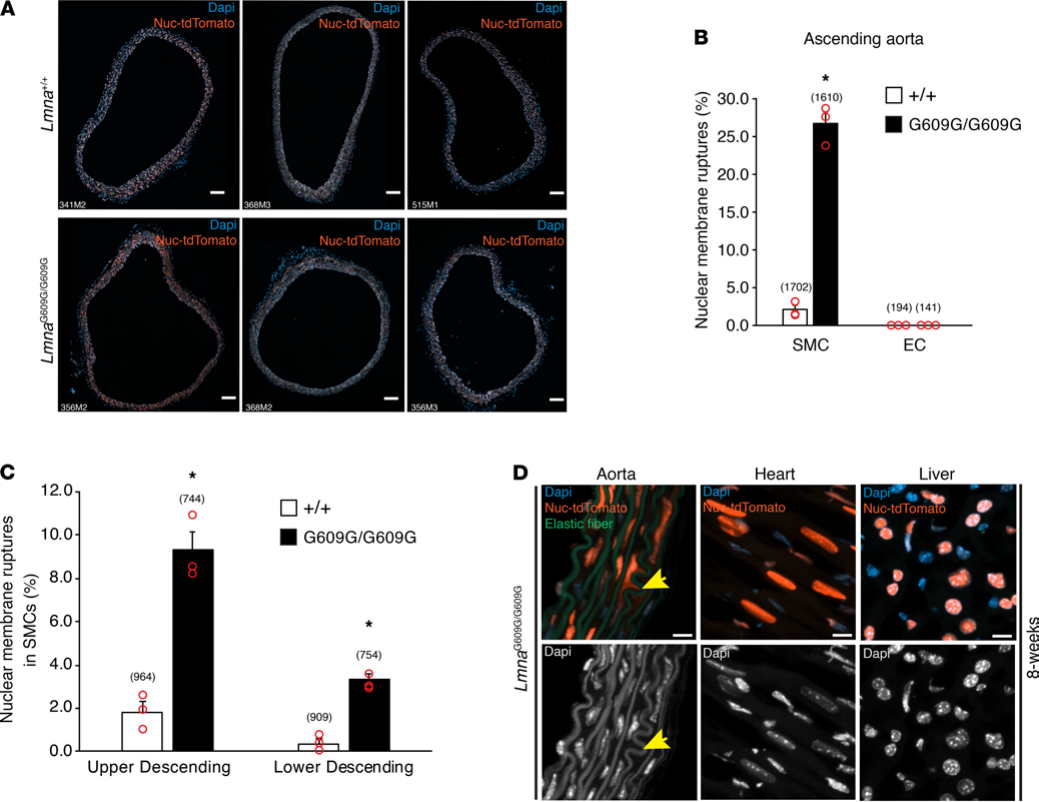
NM ruptures are frequent in aortic SMCs but absent in cardiomyocytes and hepatocytes of *Lmna*^G609G/G609G^ mice. (**A**) Fluorescence microscopy images of cross sections (10 μm–thick) of the ascending aorta from 3 8-week-old *Lmna*^+/+^ (top row) and 3 8-week-old *Lmna*^G609G/G609G^ (bottom row) mice expressing the Nuc-tdTomato transgene. The mouse IDs are shown in the bottom left-hand corner of each image. Nuc-tdTomato (orange); DAPI (blue). Scale bar: 100 μm. (**B**) Quantification of NM ruptures in SMCs and endothelial cells (EC) in the ascending aorta of 8-week-old *Lmna*^+/+^ and *Lmna*^G609G/G609G^ mice. The number of NM ruptures is reported as a percentage of total nuclei examined in individual cross sections (**A**) (mean ± SEM; Student’s *t* test; *P < 0.001). The total number of nuclei scored is shown in parentheses. (**C**) Quantification of NM ruptures in SMCs in the upper and lower descending aorta of 3 8-week-old *Lmna*^+/+^ and *Lmna*^G609G/G609G^ mice. The number of NM ruptures is reported as a percentage of total nuclei examined in individual cross sections (see Supplemental Figure 6C) (mean ± SEM; Student’s *t* test; *P < 0.001). The total number of nuclei scored is shown in parentheses. (**D**) Confocal fluorescence microscopy images of the ascending aorta, heart, and liver from an 8-week-old *Lmna*^G609G/G609G^ mouse. The colored images show DAPI (blue), elastic fibers (green), and Nuc-tdTomato (orange). To assist in visualizing the boundaries of nuclei, the DAPI stain is shown in white (bottom row). The yellow arrow points to Nuc-tdTomato outside of a nucleus in an aortic SMC. Scale bar: 10 μm.

To determine the frequency of NM ruptures in *Lmna*^G609G/G609G^ mice, NM ruptures in cross sections of the ascending thoracic aorta in 3 8-week-old *Lmna*^+/+^ mice and 3 8-week-old *Lmna*^G609G/G609G^ mice (Figure 7A) were counted and expressed relative to total SMC nuclei. The frequency of NM ruptures in aortic SMCs was significantly higher in *Lmna*^G609G/G609G^ mice than in *Lmna*^+/+^ mice (26.7% vs. 2.0%; *P* < 0.001) (Figure 7B). NM ruptures were detected in WT mice but they occurred at a very low frequency. No NM ruptures were detected in endothelial cells in either WT or *Lmna*^G609G/G609G^ mice (Figure 7B). NM ruptures were also quantified in tissue sections of the upper and lower descending aorta (Supplemental Figure 6, C and D). Consistent with the lower frequency of SMC loss in the descending thoracic aorta (12), NM ruptures were less frequent in the upper and lower descending aorta (Figure 7C). No NM ruptures were detected in the heart or liver of *Lmna*^G609G/G609G^ mice (Figure 7D).

Additional studies revealed that NM ruptures were absent in medial SMCs of *Lmna*^G609G/G609G^ mice at 6 weeks of age (0/7 mice) but were easily detectable at 7 weeks, although at a low frequency (5/5 mice). NM ruptures were also observed in older *Lmna*^G609G/+^ mice (Supplemental Figure 7, A and B).

The onset of NM ruptures in *Lmna*^G609G/G609G^ mice coincided with the onset of ultrastructural pathology in SMC nuclei (12), as judged by electron microscopy. At 4 and 6 weeks, the morphology of medial SMCs in *Lmna*^G609G/G609G^ mice was normal by electron microscopy, indistinguishable from the SMCs in WT mice. By 8 weeks, however, intranuclear tubules (the same structures described as intranuclear vesicles in ref. 12) were detected in aortic SMCs of *Lmna*^G609G/G609G^ mice (Supplemental Figure 7C). Intimal endothelial cells were entirely normal. By electron microscopy, there were no abnormalities in SMCs of the urinary bladder in 8- and 16-week-old *Lmna*^G609G/G609G^ mice, nor were there nuclear abnormalities in the heart, quadriceps, or kidney (Supplemental Figure 7D).

## Discussion

The hallmark of large artery lesions in HGPS is loss of medial SMCs (40), but for years the mechanism has been elusive. In the current study, we found that progerin expression in SMCs, combined with physiologically low levels of lamin B1, trigger NM ruptures, abnormal nuclear morphology, and SMC death. Our findings in progerin-transfected cultured SMCs and aortic SMCs of *Lmna*^G609G^ mice were concordant. In cultured SMCs, progerin expression — at levels matching those in aortas of *Lmna*^G609G/+^ mice — results in NM ruptures, DNA damage, activation of the cGAS-STING pathway, and cell death. The frequency of NM ruptures in SMCs increased with increasing levels of progerin expression. Reducing lamin B1 expression in progerin-expressing SMCs (so as to mirror the physiologically low levels of lamin B1 in aortic SMCs) and subjecting SMCs to uniaxial stretching (so as to mimic the pulsatile stretching of the aortic wall) increased the number of NM ruptures. Conversely, increasing lamin B1 expression or interfering with the transmission of cytoskeletal forces to the cell nucleus reduced NM ruptures. De Vos and colleagues (30) previously identified NM ruptures in transiently transfected human fibroblasts expressing EGFP-tagged progerin, but the incidence of NM ruptures in cells transfected with a control plasmid (e.g., EGFP-prelamin A) was not determined. The relevance of our cell culture findings to living animals was established by examining the medial SMCs in aortas of *Lmna*^G609G/G609G^ mice. In those mice, the expression of progerin in aortic SMCs, combined with very low levels of lamin B1, was accompanied by frequent NM ruptures. The onset of NM ruptures in aortic SMCs was at 7 weeks of age, preceding SMC loss.

Our studies identified nuclear lamin composition as a key factor in the vascular pathology of HGPS. The levels of progerin (which triggers NM ruptures) increased with age in the aorta of *Lmna*^G609G/+^ mice, whereas levels of lamin B1 (which protects from NM ruptures) decreased. The age-related increase in progerin in the aorta occurred despite decreasing progerin transcript levels, suggesting that progerin is a stable protein with a slow turnover rate. Lamin B1 transcripts declined with age in both WT and *Lmna*^G609G/+^ mice, but the levels of lamin B1 protein remained stable in WT mice. The preservation of lamin B1 levels in WT mice in the face of reduced transcript levels is consistent with the fact that lamin B1 has a very long half-life in mouse tissues (41, 42). However, given that lamin B1 is a long-lived protein in mice, why did aortic levels of lamin B1 fall with age in *Lmna*^G609G/+^ mice? The most likely explanation, we believe, is that progerin accelerates the turnover of lamin B1. In any case, the age-related changes in nuclear lamin composition in SMCs of *Lmna*^G609G/+^ mice — high progerin levels and low lamin B1 levels — appear to be “double trouble” for NM integrity, increasing the risk of NM ruptures. We hypothesize that the high levels of progerin and low levels of lamin B1 in SMCs increase nuclear stiffness, rendering nuclei more susceptible to damage from mechanical forces (i.e., that reduced compliance renders the NMs more susceptible to ruptures).

All of the cells in the aorta of *Lmna*^G609G^ mice (intimal endothelial cells, medial SMCs, and adventitial fibroblasts) express high levels of progerin, and all of these cells are subjected to rhythmic stretching from pulsatile blood flow, but both NM ruptures and cell death are confined to the SMCs. We suspect that differences in nuclear lamin composition explain, at least in part, why endothelial and adventitial cells are protected from NM ruptures and cell death. By immunofluorescence microscopy, lamin B1 levels are very low in the medial SMCs of *Lmna*^G609G^ mice but high in endothelial cells and adventitial cells. In cell culture studies, increasing lamin B1 expression in progerin-expressing SMCs markedly reduced nuclear shape abnormalities, NM ruptures, DNA damage, and cell death. For this reason, we suspect that the robust expression of lamin B1 in endothelial and adventitial cells protects those cells from the toxicity of progerin (i.e., NM ruptures and cell death).

Our sequential nuclear extraction experiments provided insights into how lamin B1 reduces progerin-induced NM ruptures. Progerin and lamin B1 both contain carboxyl-terminal modifications (e.g., farnesylation) that promote interaction with the inner NM (36, 43). The nuclear extraction studies revealed that increased lamin B1 expression reduced progerin’s association with the nuclear envelope, likely by limiting interactions between progerin’s farnesyl lipid with the inner NM. Consistent with this interpretation, the association of nf-Prog with NMs was far weaker than with farnesylated progerin. The reduced association of nf-Prog with NMs likely explains the inability of nf-Prog to trigger NM ruptures. We suspect that increased lamin B1 expression limits NM ruptures in progerin-expressing SMCs in the same way, by competing with progerin for binding sites at the nuclear periphery and reducing progerin’s association with the NMs.

In addition to limiting progerin’s association with the NMs, increased lamin B1 expression alters the mechanical properties of the cell nucleus. In the past, others have reported, using micropipette aspiration and nuclear strain measurements, that the cell nucleus in progerin-expressing fibroblasts is stiffer than in WT cells (44–46). In the current studies, we used AFM to quantify nuclear stiffness in progerin-expressing SMCs. Consistent with the earlier reports (44–46), we found increased stiffness of the cell nucleus in progerin-expressing SMCs, but we sought to extend this further to determine if boosting lamin B1 expression had an impact on nuclear compliance. Our AFM results were clear: increasing lamin B1 expression decreased nuclear stiffness in SMCs. Lamin B1’s capacity to decrease nuclear stiffness in SMCs was unique; nf-lamin B1, lamin A, and lamin B2 did not alter nuclear stiffness whereas knocking down lamin B1 expression increased nuclear stiffness. Remarkably, increased lamin B1 expression also decreased nuclear stiffness in progerin-expressing SMCs, indicating that the baseline levels of lamin B1 are an important factor in determining nuclear compliance (47).

Our discovery that lamin B1 decreases nuclear stiffness was initiated by studies showing that lamin B1 overexpression led to larger nuclei in adherent SMCs. Since cell spreading and cytoskeletal forces can affect nuclear size in adherent cells, we quantified the nuclear area in nonadherent SMCs that were compressed with a glass coverslip. In those studies, we observed larger nuclei in cells that overexpressed lamin B1. Lamin B1 overexpression also increased the nuclear area in progerin-expressing SMCs, but the effect was less pronounced (reflecting progerin’s ability to increase nuclear stiffness). We observed consistent findings in lamin B1–transduced fibroblasts. In those cells, we showed that lamin B1 overexpression increased nuclear area at the expense of reduced nuclear height.

The identification of NM ruptures in aortic SMCs of *Lmna*^G609G^ mice adds to our understanding of the vascular pathology in HGPS. Because NM ruptures in SMCs precede any evidence of SMC loss, it seems likely that the ruptures contribute to cell death. The expression of progerin in cultured SMCs, at levels matching those in the aorta of the *Lmna*^G609G/+^ mice, not only leads to NM ruptures but also to DNA damage and activation of the cGAS–STING pathway. The proposal that NM ruptures lead inexorably, directly or indirectly, to SMC death in *Lmna*^G609G^ mice is strengthened by recent studies of cortical neurons in the developing brain of lamin B1–deficient mouse embryos (26). In those studies, there was a clear association between NM ruptures and the death of migrating neurons within the cortical plate. Recently, NM ruptures were proposed to contribute to skeletal myopathy in *Lmna*-deficient mice (48). In those studies, NM ruptures were detected in individual muscle fibers that had been isolated by collagenase treatment and manual pipetting; however, it was unclear whether NM ruptures preceded myopathic changes.

Our current studies focused on progerin-expressing SMCs and the arterial pathology in the ascending thoracic aorta — the region with the most severe arterial disease. We would point out, however, that the disease phenotypes in mouse models of progeria (13, 49–52) are not confined to the aorta. We have not yet explored whether NM ruptures contribute to disease phenotypes in other tissues, but based on the current studies we would not be surprised if future studies found NM ruptures in other sites, particularly in tissues where conditions favor NM ruptures (i.e., high progerin, low lamin B1, and high mechanical stress).

In summary, our current studies showed that the expression of progerin, but not WT prelamin A, in cultured SMCs triggers NM ruptures, DNA damage, and reduced cell survival. We showed that both high levels of progerin and low levels of lamin B1 increase the number of NM ruptures, which are further increased in cells subjected to mechanical stretching. In aortic SMCs of *Lmna*^G609G/+^ mice, lamin B1 levels in SMCs are very low. With age, aortic lamin B1 levels in these mice decline while the levels of progerin increase. These findings, combined with our cell culture studies, led us to suspect that we would find NM ruptures in the aortic SMCs of *Lmna*^G609G^ mice. Indeed, we observed frequent NM ruptures in the medial SMCs of *Lmna*^G609G^ mice. Importantly, the onset of NM ruptures and the appearance of abnormal nuclear morphology preceded SMC loss.

## Methods

### Mice.

Mice with a targeted HGPS mutation (*Lmna*^G609G^) (11, 12) were bred with Nuc-tdTomato transgenic mice from The Jackson Laboratory (Stock 023035). The Nuc-tdTomato mouse strain was genotyped by PCR with a mutant forward primer 5′-CCAGGCGGGCCATTTACCGTAAG-3′; a WT forward primer 5′-GGAGCGGGAGAAATGGATATG-3′; and a common reverse primer 5′-AAAGTCGCTCTGAGTTGTTAT-3′ (yielding a 320-bp product for the mutant allele and a 603-bp product for the WT allele). Mice were housed in a specific pathogen-free barrier facility with a 12-hour light/dark cycle. The mice were provided pelleted mouse chow (NIH31), water *ad libitum,* and nutritional food cups (DietGel, ClearH_2_0) as required for supportive care.

### Cells.

Immortalized mouse aortic SMCs (ATCC, CRL-2797) were cultured in DMEM containing 10% FBS (HyClone), 1× nonessential amino acids, 2 mM glutamine, 1 mM sodium pyruvate, and 0.2 mg/mL G418 (12). For some experiments, SMCs were exposed to UV light (100 mJ/cm^2^) with a Stratalinker 2400 (Stratagene). To reduce lamin B1 levels, cells were transfected with 100 nM *Lmnb1* siRNA (Ambion, AM16706) using RNAiMAX (Invitrogen) according to manufacturer’s instructions.

### Dox-inducible expression of nuclear lamins in SMCs.

SMCs harboring Dox-inducible pTRIPZ expression vectors for human prelamin A and human progerin have been described previously (12). The same approach was used to generate SMCs expressing Dox-inducible constructs for lamin B1, lamin B2, nf-Prog, and nf-lamin B1 (Supplemental Table 3). The expression plasmids were constructed by InFusion cloning (Takara Bio) of cDNAs into pTRIPZ. The mouse lamin B1 cDNA was amplified from pCMV6-*Lmnb1* (Origene, MC219388) with forward primer 5′-TACCGGTCCGGAATTATGGCGACCGCGAC-3′ and reverse primer 5′-ATACTCTAGAGCGGCTCACATAATGGCACA-3′. The human lamin B2 cDNA was amplified from pCMV6-*LMNB2* (Origene, SC106163) with forward primer 5′-ATGCGTGGACCTGGAGAAA-3′ and reverse primer 5′-GTAGCCCCTTGAATTTCACATCACGTAGCAGCCTCTTGA-3′. A nf-Prog cDNA was created by changing the C-terminal –CSIM signal sequence in pCMV-XL5-progerin (12) to –SSIM using the QuickChange Lightning mutagenesis kit (Agilent) with forward primer 5′-CAGAGCCCCCAGAACTCCAGCATCATGTAATCT-3′ and reverse primer 5′-AGATTACATGATGCTGGAGTTCTGGGGGCTCTG-3′. A nf-lamin B1 cDNA was created by changing the C-terminal –CAIM signal sequence in pCMV6-*Lmnb1* (Origene, MC219388) to –SAIM with forward primer 5′-GTCAGATCGCACCGGATGGCGACCCGCGA-3′ and reverse primer 5′-GGCTCACATAATGGCACTGCTTTTATTGGATGCTC-3′. All plasmids were verified by DNA sequencing. Packaging of lentivirus and cell transduction were performed by the UCLA Vector Core. Transduced cells were selected with 1.5 μg/mL puromycin for 2 weeks; clones were isolated by limiting dilution.

### Constitutive expression of RFP and nuclear lamins in SMCs.

SMCs expressing GFP with a nuclear localization signal (NLS-GFP), KASH2-GFP, and extKASH2-GFP have been described previously (12, 26). SMCs expressing NLS-RFP, prelamin A, and progerin were generated by transducing SMCs with pCDH expression plasmids (Supplemental Table 3). The pCDH-NLS-RFP plasmid was created by excising the GFP cDNA in pCDH-NLS-GFP (26) with *Sma*I and *Not*I and replacing it with the RFP cDNA from TurboRFP-H2B (Addgene, 58047) by InFusion cloning. The RFP cDNA was amplified with forward primer 5′-GCGTAAGGTTCCCGGTATGAGCGAGCTGATCAAGGAGA-3′ and reverse primer 5′-CAGATCCTTGCGGCCTTATCTGTGCCCCAGTTTGC-3′. The prelamin A and progerin cDNAs were subcloned into *Eco*RI and *Not*I sites of pCDH by In-Fusion cloning. Human prelamin A was amplified with forward primer 5′-GCTAGCGAATTATGGAGACCCCGTCCCAGC-3′ and reverse primer 5′-GATCCTTGCGGCCTTACATGATGCTGCAGT-3′; and human progerin was amplified with forward primer 5′-GCTAGCGAATTATGGAGACCCCGTCCCAGC-3′ and reverse primer 5′-CAGATCCTTGCGGCCTTACATGATGCTGCA-3′. All plasmids were verified by DNA sequencing. Packaging of lentivirus and cell transduction was performed by UCLA’s Vector Core. Transduced cells were selected with 3 μg/mL blasticidin (Thermo Fisher Scientific) for 2 weeks; clones were isolated by limiting dilution.

### Measurement of NM ruptures in live SMCs.

SMCs stably expressing GFP or RFP in the nucleus were seeded into 2-well chamber slides with glass coverslip bottoms (Thermo Fisher Scientific) and cultured in DMEM with 10% FBS (as described earlier). Dox was added to induce nuclear lamin expression and incubated for 24 hours before examination by microscopy. The cell culture chamber slide was mounted into a CO_2_ and temperature-controlled stage on a Zeiss LSM 800 confocal laser-scanning microscope controlled by Zen Blue 2.3 software (all from Zeiss). The cells were visualized for 24 hours at 37°C and 5% CO_2_ with a Plan-Apochromat 20×/0.8 NA objective. Images of a 3 × 3 tiled field (~1.3 mm^2^) were captured every 10 minutes. Differential interface contrast (DIC) was acquired with the transmitted light detector (T-PMT; Zeiss) at the same time as the GFP signal. Composite images of 10 *z*-sections (1 μm sections) were generated and analyzed for NM ruptures. The number of cells with a NM rupture, defined by the escape of GFP (or RFP) in the cytoplasm, was divided by the total number of cells in a field.

### Measurement of NM ruptures in stretched SMCs.

SMCs stably expressing GFP were seeded onto PDMS membranes and cultured for 48 hours. The membranes were clamped into a custom-built biaxial cell stretching device (12) and stretched 2 mm at 0.5 Hz for 2 hours. The membranes were fixed with 4% paraformaldehyde (PFA; MilliporeSigma) and processed for confocal laser-scanning microscopy. Images from random locations were acquired and scored for NM ruptures. A minimum of 200 cells were scored by 2 trained observers blinded to sample identity.

### Measurement of NM ruptures in aortic sections.

Mice expressing the Nuc-tdTomato transgene were perfused with 3% PFA in PBS and tissues postfixed in the same solution at 4°C. Frozen tissue sections (10 μm–thick) were stained with DAPI and mounted in Antifade (Invitrogen). Images were captured on a Zeiss LSM 800 laser-scanning confocal microscope controlled by Zen Blue 2.3 software using a Plan-Apochromat 20×/0.8 NA objective. Maximum image projections were generated from scans of the tdTomato and DAPI channels, combined with the autofluorescence signal in the GFP channel. The latter was used to identify the elastic fibers in the media layer. A NM rupture was defined as the appearance of tdTomato fluorescence outside of a nucleus, as judged by DAPI staining. If tdTomato fluorescence was present between 2 adjacent nuclei, this was counted as a single NM rupture. NM ruptures were counted and expressed relative to the total number of nuclei examined.

### Measurement of phosphorylated-STING in static and stretched SMCs.

SMCs expressing prelamin A or progerin were seeded onto PDMS membranes and cultured for 48 hours. Membranes were stretched for 3 hours (2 mm at 0.5 Hz), fixed with 4% PFA, and processed for immunocytochemistry. Cells were incubated with antibodies against phosphorylated-STING (Cell Signal Technology) and LAP2β, and bound antibodies detected with species-specific fluorescent-labeled antibodies (Supplemental Table 1). Fluorescence microscopy was performed on a Zeiss LSM 800 laser-scanning microscope. Quantification of cells with positive phosphorylated-STING staining was performed by 2 trained observers blinded to sample identity. A minimum of 200 cells were scored per group.

### Sequential extraction of SMC nuclei.

SMC nuclei were extracted as described by Nmezi and colleagues (38). The sequential extraction of nuclei (with low and high salt, detergent, and urea), and quantification of nuclear lamins in the soluble extracts, provides a measure of the association of nuclear lamins with the nuclear envelope. SMCs in 100 mm tissue culture dishes were collected by scraping into 3 mL of ice-cold PBS and centrifugation at 10,000 *g* for 30 seconds. The cell pellet was resuspended in 1 mL of lysis buffer (ice-cold PBS, 0.1% IGEPAL from Sigma-Aldrich, and protease inhibitors from Sigma-Aldrich) and the lysate centrifuged at 10,000 *g* for 1 minute at 4°C. The pellet was resuspended in 250 μL of nuclear isolation buffer (10 mM HEPES pH 7.4; 2 mM MgCl_2_; 25 mM KCl; 250 mM sucrose; 1mM DTT; and protease inhibitors), and 50 μL (“nuclei” fraction) was set aside for analysis by western blotting. The remainder was sonicated on ice (five 2-second pulses), centrifuged at 20,000 *g* for 5 minutes at 4°C, and the supernatant (“sonicate” fraction) collected. The pellet was resuspended in 150 μL of nuclear extraction buffer (20 mM HEPES pH 7.4 and protease inhibitors) containing 0.2 M NaCl, and incubated for 20 minutes with end-over-end rotation. The sample was centrifuged at 200,000 *g* for 10 minutes at 4°C and the supernatant (“0.2 M NaCl” fraction) collected. The extraction procedure was repeated with 150 μL of 0.5 M NaCl, 2% Triton X100, 4 M urea, and 8 M urea (all in nuclear extraction buffer). Protein extracts were stored at –80°C until analysis.

### Measurement of nuclear area in nonadherent cells.

Dox was added to SMCs to induce nuclear lamin expression, and then stained with Hoechst 33342 (5 μg/mL) for 5 minutes in PBS. The cells were washed 3 times with PBS and detached with trypsin. The cells were resuspended in culture media and diluted to 1 × 10^6^ cells per mL. A 5-μL aliquot of stained cells was placed into a glass coverslip-bottom dish (Thermo Fisher Scientific) and allowed to settle for 5 minutes. Images of cell nuclei were recorded with a Zeiss LSM 800 confocal microscope before and after placing a 12 mm coverslip (Fisher Scientific) on top of the cells. The nuclear area was measured with ImageJ (NIH) software.

### Measuring nuclear stiffness by AFM.

SMCs in 35 mm glass coverslip-bottom dishes (World Precision Instruments) were incubated with Dox for 24 hours to induce nuclear lamin expression. Nuclear stiffness (Young’s modulus) in adherent cells was measured on a NanoWizard 4a Bioscience AFM (JPK Instruments) coupled with a Zeiss LSM5 confocal fluorescence microscope (53, 54). A spherical AFM tip with a radius of 500 nm and 0.2 N/m spring constant cantilever (Nanotools, B500-CONT) was placed on top of the cell over the nucleus, identified by staining DNA with Hoechst 33342. Three different areas of the nucleus were sampled with a 2-nN maximum set point. The 3 measurements were used to calculate the average Young’s modulus for a single cell. All measurements were performed at 37° C in HEPES-buffered culture medium. The Young’s modulus was measured in 25 randomly selected cells for each group, and the force curves analyzed with JPK Data Processing software (JPK Instruments). The Young’s modulus was calculated with the Hertz model for a spherical tip and applied to fit the slopes of the approach curve.

### Statistics.

Statistical analyses were performed with GraphPad Prism software. Experimental groups were analyzed by unpaired 2-tailed Student’s *t* test, or 1-way and 2-way ANOVA with Tukey’s multiple comparisons test. Statistical significance was considered when the P value was less than 0.05. Red circles in bar graphs show the average values of independent experiments or values for individual animals.

### Study approval.

All animal studies were approved by UCLA’s Animal Research Committee.

## Author contributions

PHK, SGY, and LGF designed the research studies. PHK, NYC, YT, PJH, TAW, JLCF, and LGF performed the experiments. NKG and PHK conducted the parallel microfiltration studies. PHK, SGY, and LGF wrote the first draft of the manuscript. PHK, NYC, PJH, YT, TAQ, JLCF, NKG, ACR, SGY, and LGF edited the manuscript.

## Supplementary Material

Supplemental data

Supplemental video 1

Supplemental video 2

Supplemental video 3

Supplemental video 4

## Version 1 08/23/2021

Electronic publication
