# Supplementary material for: Nuclear membrane ruptures underlie the vascular pathology in a mouse model of Hutchinson-Gilford progeria syndrome
Source: JCI Insight. 2021 Aug 23;6(16):e151515. doi: 10.1172/jci.insight.151515 (PMC8409987; doi:10.1172/jci.insight.151515)

Supplementary Materials

Nuclear membrane ruptures underlie the vascular pathology in a mouse model of Hutchinson-Gilford progeria syndrome

Materials and Methods.

Fig. S1. Progerin causes NM ruptures in SMCs.

Fig. S2. Disrupting the LINC complex reduces NM ruptures in Prog-SMCs.

Fig. S3. Nonfarnesylated progerin is bound less tightly to nuclear membranes.

Fig. S4. Lamin B1 decreases nuclear stiffness.

Fig. S5. Progerin and lamin B1 proteins levels change with age in the aorta of *Lmna*$^{G609G/+}$ mice.

Fig. S6. NM ruptures in SMCs of the ascending thoracic aorta.

Fig. S7. NM ruptures and intranuclear membranous tubules in aortic SMCs of *Lmna*$^{G609G/+}$ mice.

Table S1. Antibodies used for western blotting and immunohistochemistry.

Table S2. Quantitative RT-PCR primers.

Table S3. Plasmid constructs.

Supplementary Materials and Methods

Dox-inducible expression of lamin B1 in mouse embryonic fibroblasts (MEFs). MEFs were transduced with the pTRIPZ-*Lmnb1* construct by UCLA's Vector Core. Transduced cells were selected with 3μg/ml puromycin for two weeks; clones were isolated by limiting dilution.

Quantitative real time-PCR. Total RNA was isolated and treated with DNase I (Ambion) according to the manufacture's recommendation. RNA was reverse-transcribed with random primers with SuperScript III cDNA Synthesis Kit (Invitrogen). cDNA samples were diluted in nuclease-free water and stored at –80° C. RT-PCR reactions were performed on a 7900 Fast Real-Time PCR system (Applied Biosystems) with SYBR Green PCR Master Mix (Bioline). Transcript levels were calculated by the comparative cycle threshold method and normalized to cyclophilin A expression. All oligonucleotide primers are listed in Table S2.

Western blotting. Urea-soluble protein extracts from cells and tissues were prepared as described (12). Proteins were size-fractionated on 4–12% gradient polyacrylamide Bis-Tris gels (Invitrogen) and transferred to nitrocellulose membranes. The membranes were blocked with Odyssey Blocking solution (LI-COR Bioscience, Lincoln, NE) for 1 h at RT and incubated with primary antibodies at 4° C overnight. After washing the membranes with PBS containing 0.2% Tween-20, they were incubated with infrared dye (IR)–labeled secondary antibodies at RT for 1 h. The IR signals were quantified with an Odyssey infrared scanner (LI-COR Biosciences). The antibodies and concentrations are listed in Table S1.

Immunofluorescence microscopy. Cells on coverslips or tissue sections (6–10-μm-thick) on glass slides were fixed with 4% paraformaldehyde in PBS and permeabilized with 0.2% Triton. The cells were processed for immunofluorescence microscopy as (12). The antibodies and antibody concentrations are listed in Table S1. Confocal fluorescence microscopy images were

obtained with a Zeiss LSM 800 laser-scanning microscope and images along the *z*-axis were processed by Zen Blue 2.3 software to generate maximum image projections.

Electron microscopy. Mice were perfused *in situ* with PBS followed by ice-cold fixative (2.5% glutaraldehyde, 3% paraformaldehyde in PBS). Tissue samples were processed as described previously (12). Images were acquired with an FEI T12 transmission electron microscope set to 120 kV accelerating voltage and a Gatan 2K × 2K digital camera (Electron Imaging Center).

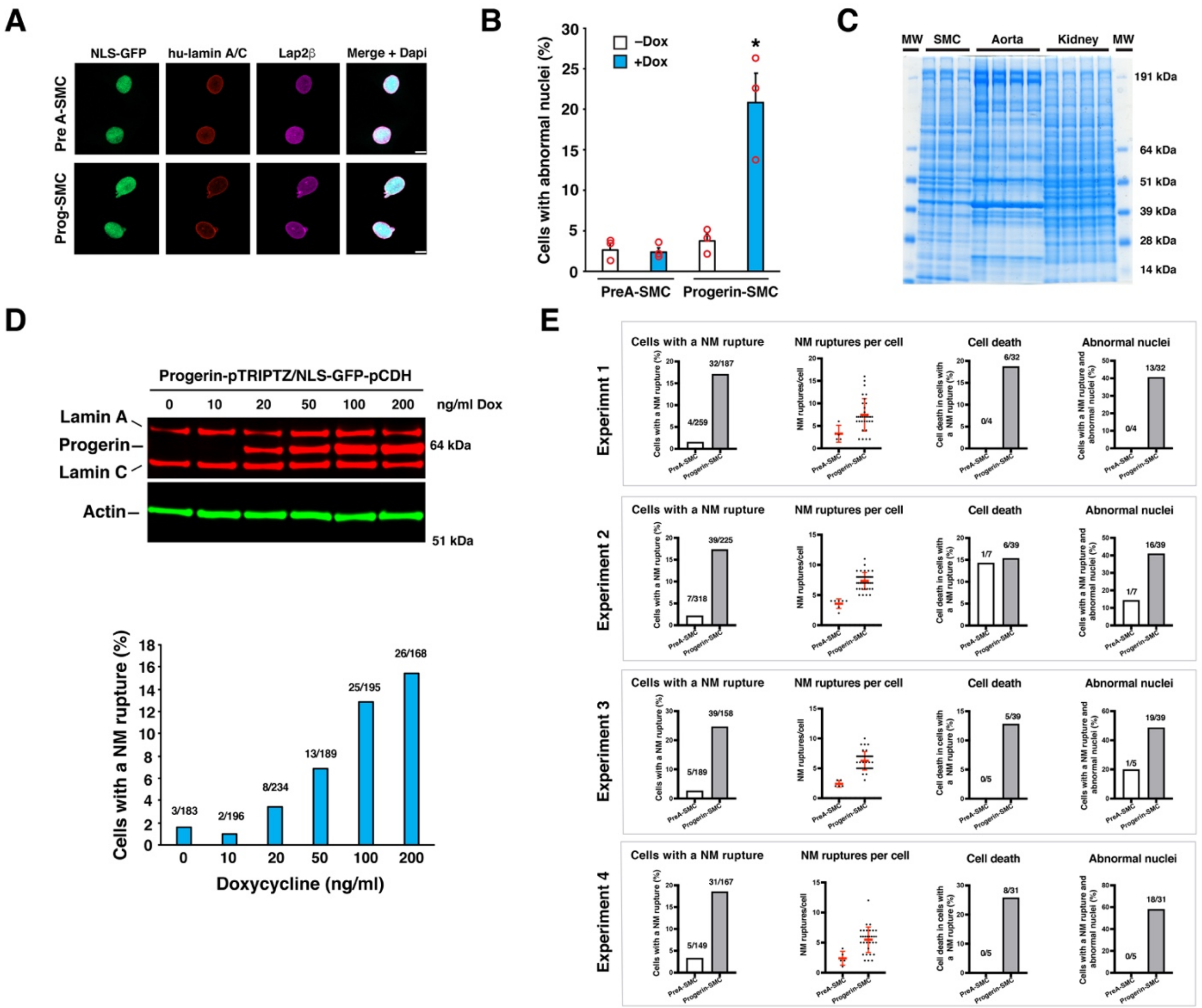

Fig. S1. Progerin causes NM ruptures in SMCs. (A) Confocal fluorescence microscopy images showing the expression of human prelamin A and human progerin in SMCs that express Nuc-GFP (*green*). Cells were stained with antibodies against human lamin A (*red*) and lap2β (*magenta*); nuclei were stained with Dapi (*blue*). Scale bar, 10 µm. **(B)** Bar graph showing that progerin increases abnormally shaped nuclei in fixed SMCs (mean ± SEM, $n = 3$ experiments; t-test, $*p < 0.02$). Progerin and prelamin A expression (*blue*) were induced with doxycycline (Dox). *Red* circles show the average values in individual experiments. **(C)** Coomassie Blue–stained SDS-PAGE gel used to quantify total protein for the western blot in Figure 1A. MW, molecular weight. **(D)** Progerin causes a dose-dependent increase in NM ruptures in SMCs. (Top) Western blot showing increasing progerin expression with increasing amounts of Dox. Actin was measured as a loading control. (Bottom) Bar graph showing dose-dependent increase in NM ruptures in SMCs. The ratios above each bar show the numbers of cells with a NM rupture over the total number of cells examined. **(E)** Characterization of NM ruptures in PreA-SMCs and Progerin-SMCs, as judged by time-lapse microscopy ($n = 4$ experiments). Bar graphs show the percentage of cells with a NM rupture; the number of NM ruptures per cell; the percentage of cells with a NM rupture that die; and the percentage of cells with a NM rupture and abnormal shaped nuclei. A total of 915 PreA-SMCs and 735 Prog-SMCs were analyzed.

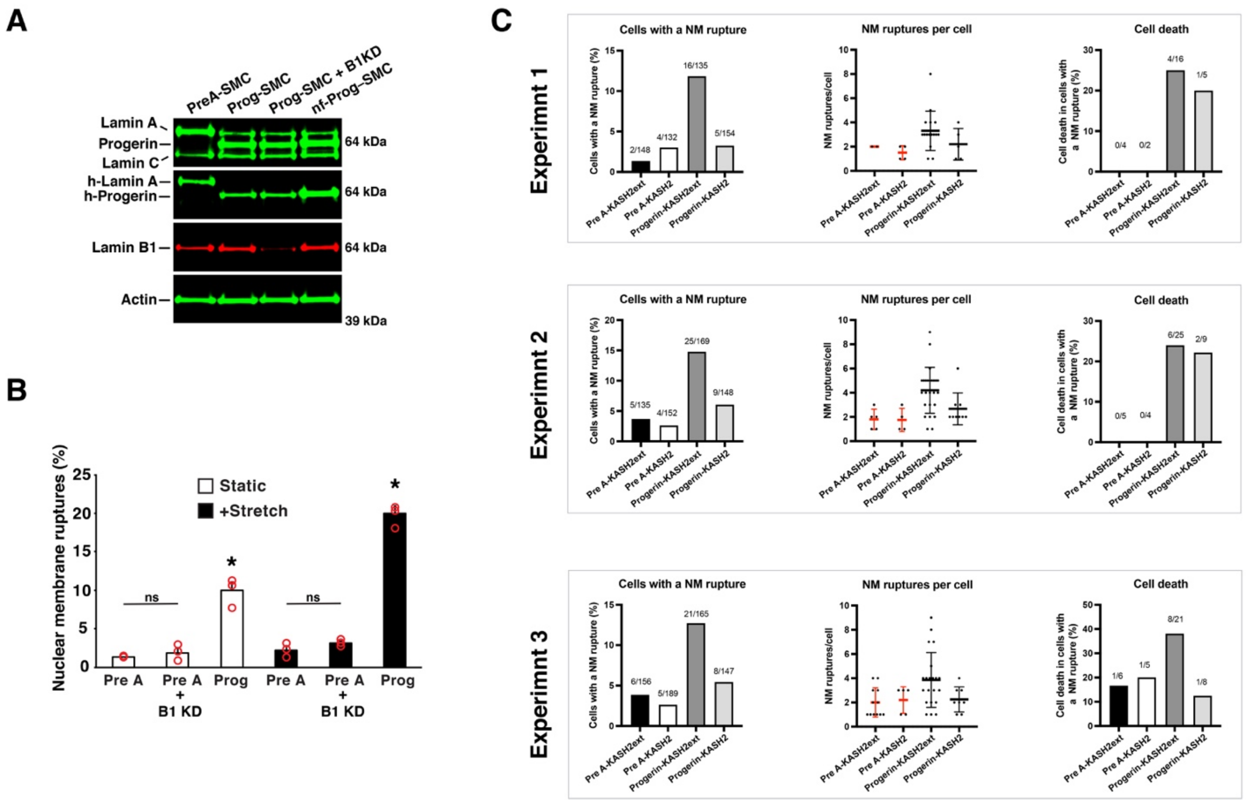

Fig. S2. Disrupting the LINC complex reduces NM ruptures in Prog-SMCs. (A) Western blot showing the expression of prelamin A, progerin, and nonfarnesylated progerin (all human) in SMCs, and the effects of lamin B1 siRNA knockdown (B1KD) in Prog-SMCs. Actin was measured as a loading control. **(B)** Bar graph showing that lamin B1 knockdown (B1 KD) does not increase NM ruptures in PreA-SMCs under either static (*white* bars) or stretched (*black* bars) conditions. Values compared to NM ruptures in static PreA-SMCs (mean ± SEM, $n = 3$ experiments; two-way ANOVA, $*p < 0.0001$). ns, non-significant. *Red* circles show average values in individual experiments. **(C)** Characterization of NM ruptures in PreA-SMC and Prog-SMCs in the presence of either KASH2 or KASH2ext, as judged by time-lapse microscopy ($n = 3$ experiments). Bar graphs show the percentage of cells with a NM rupture; the number of NM ruptures per cell; and the percentage of cells with a NM rupture that die. The total number of cells scored in each experiment is shown in the bar graph on the left.

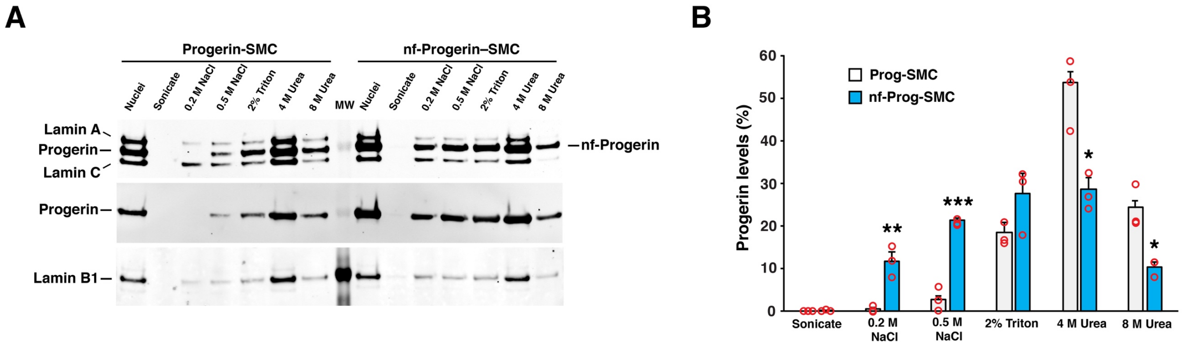

Fig. S3. Nonfarnesylated progerin is bound less tightly to nuclear membranes. (A) Western blot showing the extraction profiles for progerin and nonfarnesylated progerin expressed in SMCs. Nuclei isolated from Prog-SMCs and nf-Prog-SMCs were sequentially extracted with 0.2 M NaCl, 0.5 M NaCl, 2% Triton, 4 M urea, and 8 M urea. The supernatants from each extraction were analyzed by western blotting with antibodies against lamin A/C (to detect all A-type lamins), human lamin A (to detect progerin and nf-progerin), and lamin B1. **(B)** Bar graph comparing the extraction profiles for progerin (*white*) and nf-progerin (*blue*) (mean ± SEM, $n = 3$ experiments; t-test, $*p < 0.02$, $**p < 0.01$, $***p < 0.001$). *Red* circles show average values in individual experiments.

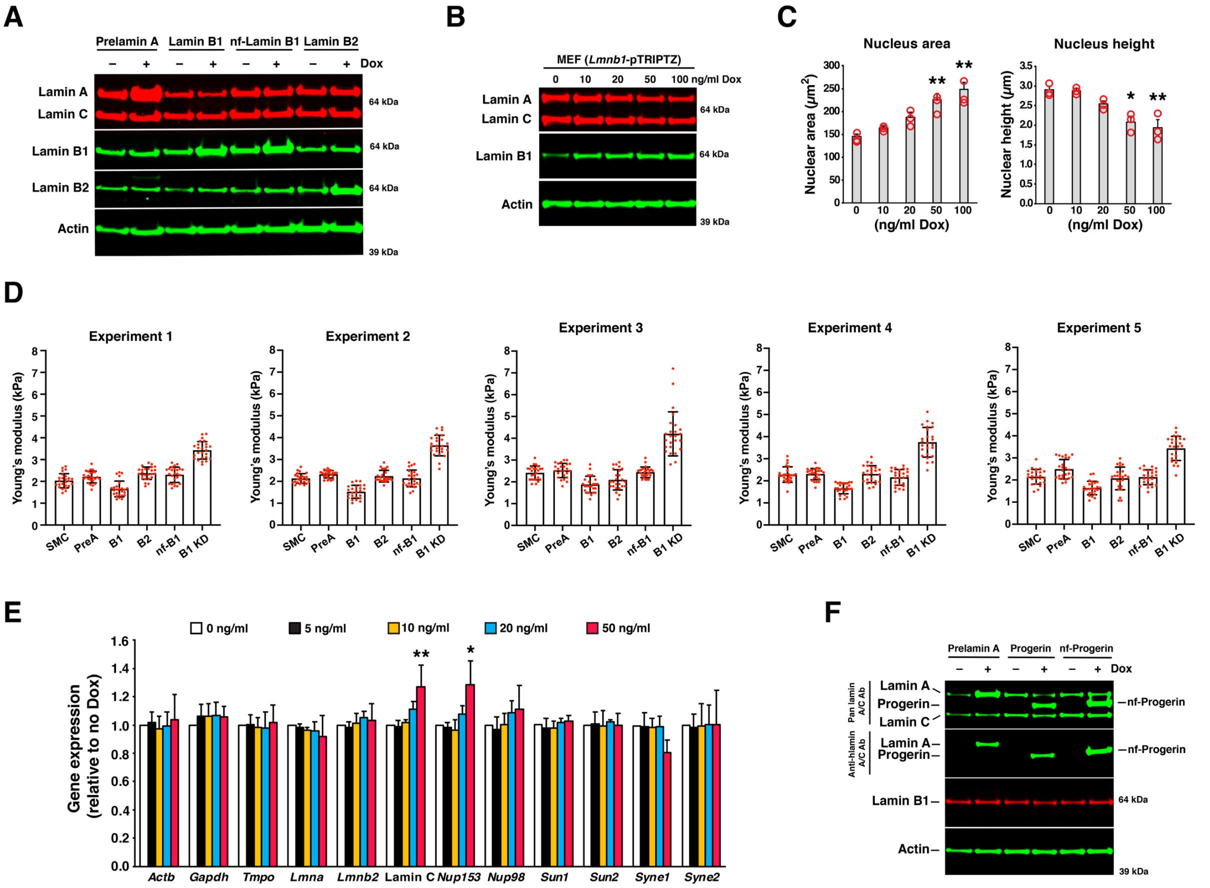

Fig. S4. Lamin B1 decreases nuclear stiffness. (A) Western blot showing the expression of prelamin A, lamin B1, nonfarnesylated lamin B1 (nf-lamin B1), and lamin B2 in SMCs. Lamin expression was induced with Dox. Actin levels were measured as a loading control. **(B)** Western blot showing inducible expression of lamin B1 in mouse embryonic fibroblasts (MEFs). Lamin B1 expression was induced with Dox. Actin levels were measured as a loading control. **(C)** Bar graphs showing the effects of lamin B1 on nuclear size in MEFs. Lamin B1 expression was induced in adherent MEFs with Dox (0–100 ng/ml) for 48 h. The cells were imaged by confocal fluorescence microscopy, and the nucleus area and height determined (mean ± SEM, n = 3 experiments; ANOVA, *p < 0.01, **p < 0.002). *Red* circles show the average values for independent experiments. **(D)** The effects of prelamin A (PreA), lamin B1 (B1), lamin B2 (B2), nf-lamin B1 (nf-B1), and lamin B1 knockdown (B1 KD) on nuclear stiffness in SMCs were measured by AFM 48 h after changing nuclear lamin expression. Each *red* dot represents an individual cell. Twenty-five cells were examined for each group. Mean ± SD. **(E)** Bar graph comparing the effects of lamin B1 expression on gene expression (mean ± SEM, n = 3 experiments). Comparisons made to SMCs not induced with Dox (*white* bar); ANOVA, *p < 0.02, **p < 0.01. **(F)** Western blot showing the expression of human prelamin A, human progerin, and human nonfarnesylated–progerin (nf-Progerin) in SMCs. A "pan lamin AC antibody" was used to detect mouse and human A-type lamins; a human lamin A–specific antibody was used to detect human lamin A and progerin. Actin was measured as a loading control.

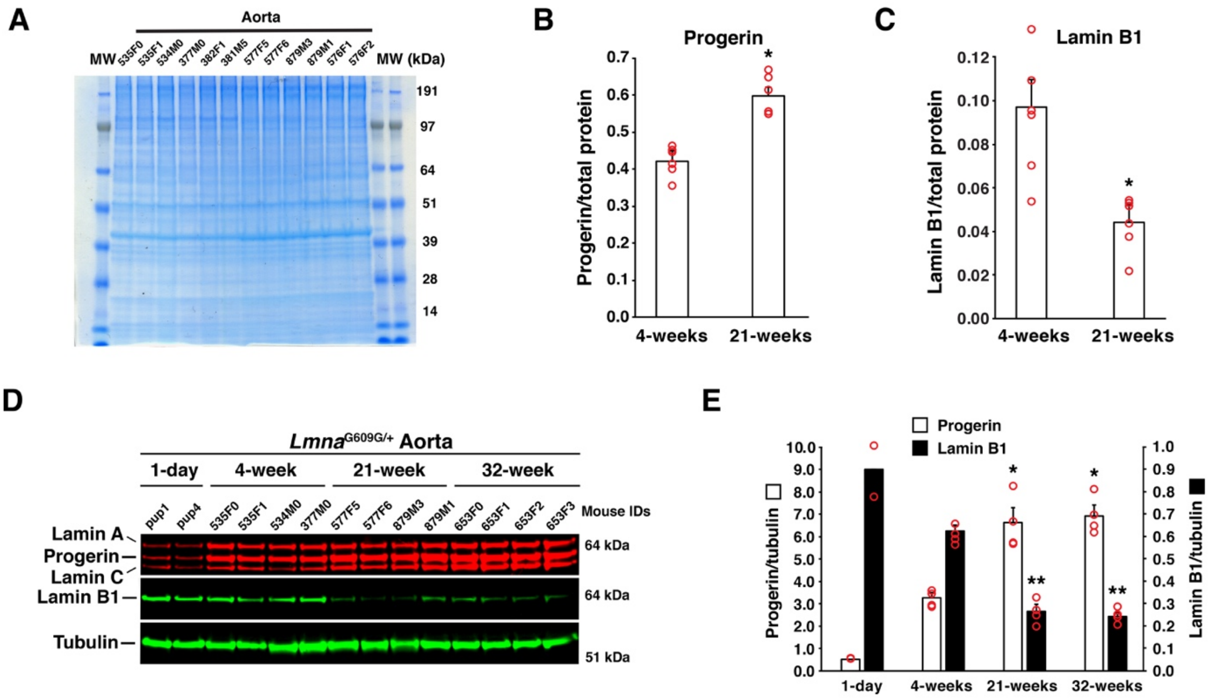

Fig. S5. Progerin and lamin B1 proteins levels change with age in the aorta of *Lmna*$^{G609G/+}$ mice. (A) Coomassie Blue–stained gel corresponding to the western blot in Figure 5A. MW, molecular weight. **(B)** Bar graph showing progerin levels, relative to total protein, for the western blot in Figure 5A (mean ± SEM, $n = 6$ mice/group; t-test, $*p < 0.001$). *Red* circles show values for individual animals. **(C)** Bar graph showing lamin B1 levels, relative to total protein, for the western blot in Figure 5A (mean ± SEM, $n = 6$ mice/group; t-test, $*p < 0.005$). *Red* circles show values for individual animals. **(D)** Western blot comparing the expression of progerin and lamin B1 in the aorta from 1-day-old, and 4-, 21-, and 32-week–old *Lmna*$^{G609G/+}$ mice. Thoracic aortas (2 or 4 mice per group) were extracted and analyzed as described in Figure 5A. IDs are shown above each sample. **(E)** Bar graph showing the expression of progerin (*white* bars) and lamin B1 (*black* bars), relative to tubulin, for the western blot in panel D. Shown are mean ± SEM ($n = 4$ mice/group), except for the 1-day old mice ($n = 2$). The values for the 21- and 32-week–old mice are compared to 4-week–old mice by ANOVA ($*p < 0.001$, $**p < 0.0001$). *Red* circles show the values for individual animals.

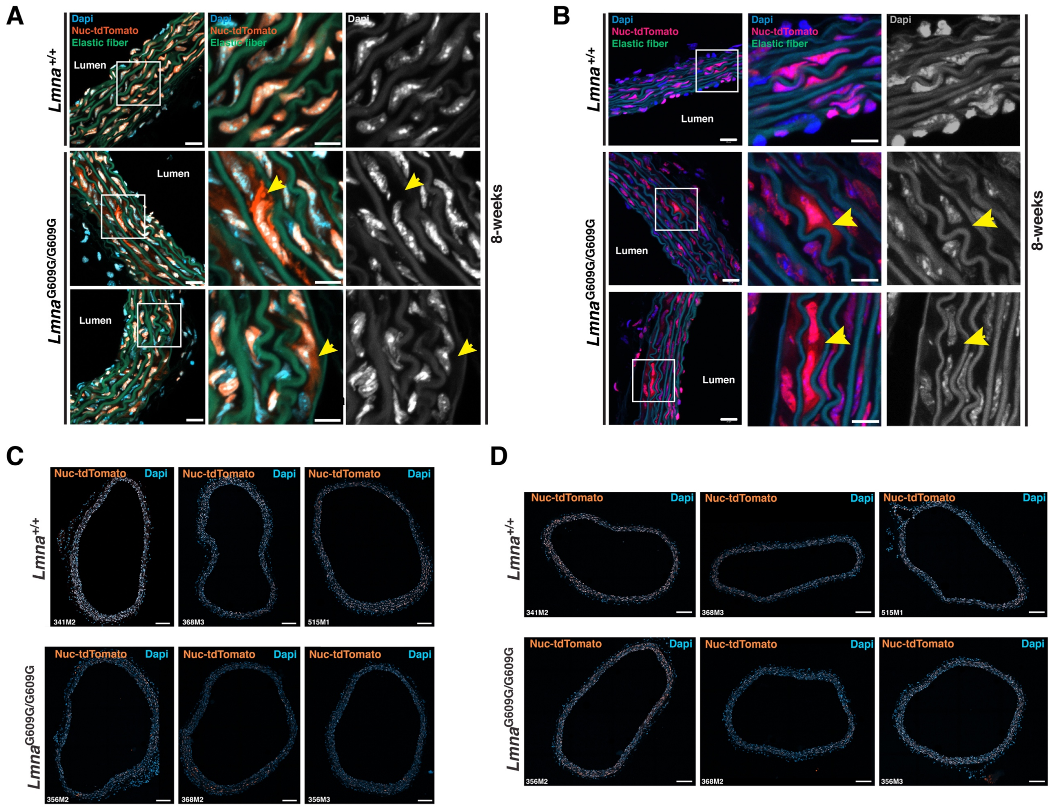

Fig. S6. NM ruptures in SMCs of the ascending thoracic aorta. (A, B) Confocal fluorescence microscopy images of the ascending aorta from 8-week–old *Lmna*$^{+/+}$ and *Lmna*$^{G609G/G609G}$ mice. The boxed regions are shown at higher magnification in the middle and far-right columns. The colored images show Dapi (*blue*), elastic fibers (*green*), and Nuc-tdTomato (*orange* or *red*). The *yellow* arrows point to Nuc-tdTomato outside of SMC nuclei. To help visualize the boundaries of nuclei, the Dapi stain (*white*) is shown by itself in the far-right column. Scale bars: 20 µm (left column); 10 µm (middle column). **(C, D)** Fluorescence microscopy images of cross sections (10-µm-thick) of the upper (C) and lower (D) descending aorta from three 8-week–old *Lmna*$^{+/+}$ (top row) and three *Lmna*$^{G609G/G609G}$ (bottom row) mice. NM ruptures were quantified in these tissue sections (Fig. 7). The mouse IDs are shown in the bottom left hand corner of each image. Nuc-tdTomato (*orange*); Dapi (*blue*). Scale bars, 100 µm.

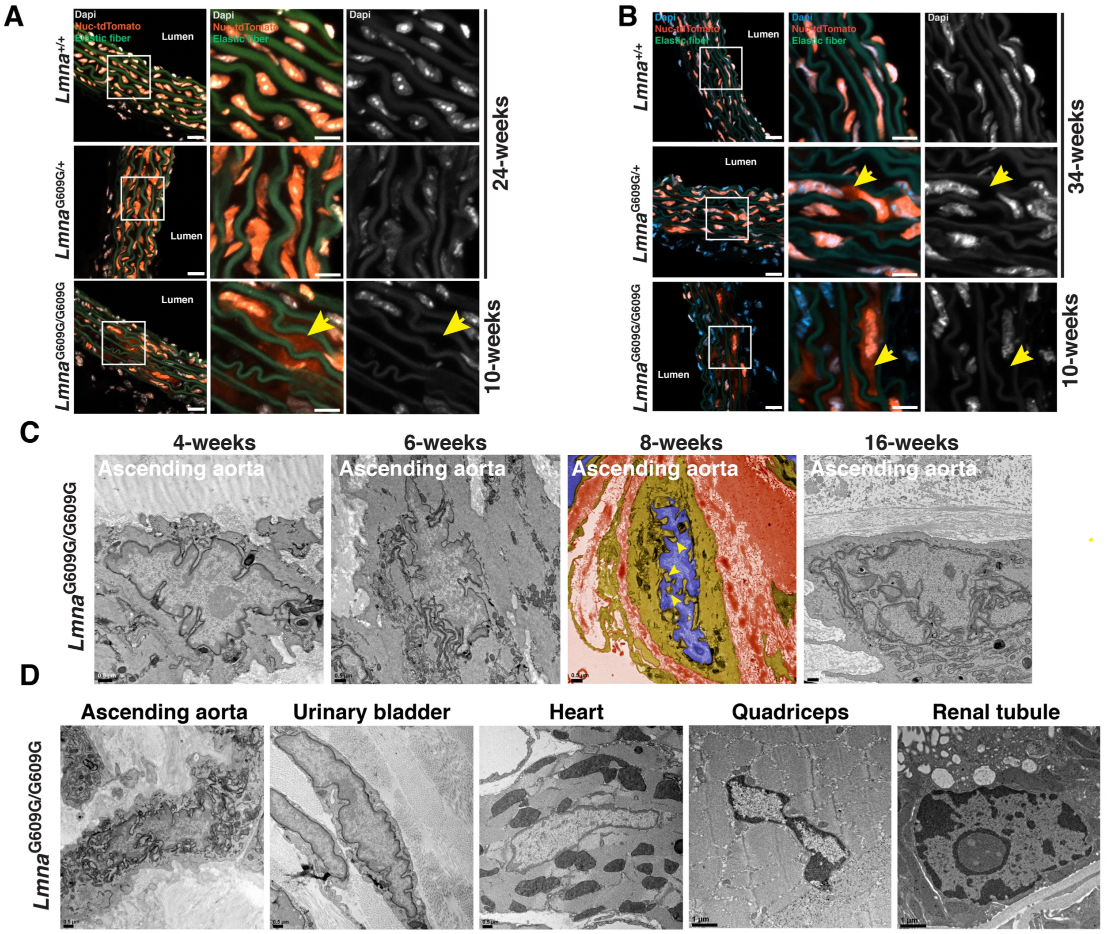

Fig. S7. NM ruptures and intranuclear membranous tubules in aortic SMCs of *Lmna*$^{G609G/+}$ mice. (A) Confocal fluorescence microscopy images of the ascending aorta from 24-week–old *Lmna*$^{+/+}$ and *Lmna*$^{G609G/+}$ mice and a 10-week–old *Lmna*$^{G609G/G609G}$ mouse. The boxed regions are shown at higher magnification on the right. The colored images show Dapi (*white*), elastic fibers (*green*), and Nuc-tdTomato (*orange*). The *yellow* arrow (middle column) points to Nuc-tdTomato outside of an SMC nucleus in the *Lmna*$^{G609G/G609G}$ mouse. To help visualize the boundaries of nuclei, the Dapi stain (*white*) is shown in the right column. Scale bars: 20 µm (left column); 10 µm (middle column). **(B)** Confocal fluorescence microscopy images of the ascending aorta from 34-week–old *Lmna*$^{+/+}$ and *Lmna*$^{G609G/+}$ mice and a 10-week–old *Lmna*$^{G609G/G609G}$ mouse. The boxed regions are shown at higher magnification to the right. Images were generated exactly as described in panel A except that the Dapi signal is colored *blue*. *Yellow* arrows point to Nuc-tdTomato outside of nucleus in the 34-week–old *Lmna*$^{G609G/+}$ mouse and the 10-week–old *Lmna*$^{G609G/G609G}$ mouse. Scale bars: 20 µm (left column); 10 µm (middle column). **(C)** Representative electron micrographs of SMC nuclei in the ascending aorta from 4-, 6-, 8-, and 16-week–old *Lmna*$^{G609G/G609G}$ mice. The image from the 8-week-old mouse was colored to make it easier to identify the cell nucleus (*blue*), cytoplasm (*tan*), and connective tissue (*reddish-brown*). *Yellow* arrowheads point to intranuclear tubules. Scale bars are shown in the images. **(D)** Representative electron micrographs of nuclei in the ascending aorta, urinary bladder, heart, quadriceps, and renal tubule from a 16-week–old *Lmna*$^{G609G/G609G}$ mouse. Scale bars are shown in the images.

Table S1. Antibodies used for western blotting and immunohistochemistry.

Antibody Description	Species	Source	Catalog #	Use	Dilution
β-actin	Goat	Santa Cruz Biotech	SC1616	WB, IF	1:3000
GFP	Rabbit	ThermoFisher	A11122	WB, IF	1:1000
Human lamin A	Mouse	Millipore	MAB3211	WB, IF	1:1500
Lamin A/C	Mouse	Santa Cruz Biotech	SC376248	WB, IF	1:1500
Lamin B1	Goat	Santa Cruz Biotech	SC6217	WB, IF	1:1500
Lamin B2	Mouse	Invitrogen	332100	WB, IF	1:1000
LAP2β	Mouse	BD Pharmingen	611000	IF	1:2000
Phospho-H2AX	Mouse	Millipore	05-636	WB, IF	1:3000
Phospho-STING (Ser365)	Rabbit	Cell Signaling Technology	72971S	IF	1:1000
Phospho-p53(Ser15)	Rabbit	Cell Signaling Technology	9284	WB, IF	1:3000
Tubulin	Rat	Novus Bio	NB600-506	WB	1:3000
Anti-rabbit IR800	Donkey	LI-COR	926-32213	WB	1:10000
Anti-goat IR800	Donkey	LI-COR	926-32214	WB	1:10000
Anti-rat IR800	Donkey	ThermoFisher	SA5-10032	WB	1:5000
Anti-mouse IR800	Donkey	ThermoFisher	SA5-10172	WB	1:5000
Anti-rabbit IR680	Donkey	LI-COR	926-32221	WB	1:5000
Anti-rat IR680	Goat	LI-COR	925-68076	WB	1:5000
Anti-goat IR680	Donkey	LI-COR	926-68074	WB	1:5000
Anti-mouse IR680	Donkey	ThermoFisher	SA5-10170	WB	1:5000
Anti-mouse Alexa 488	Donkey	Invitrogen	A21202	IF	1:2000
Anti-rabbit Alexa 488	Donkey	Invitrogen	A21206	IF	1:2000
Anti-goat Alexa 488	Donkey	Invitrogen	A11055	IF	1:2000
Anti-goat Alexa 555	Donkey	Invitrogen	A21432	IF	1:2000
Anti-rabbit Alexa 555	Donkey	Invitrogen	A31572	IF	1:200
Anti-rabbit Alexa 568	Donkey	Invitrogen	A10042	IF	1:2000
Anti-mouse Alexa 568	Donkey	Invitrogen	A10037	IF	1:2000

Anti-mouse Alexa 647	Donkey	Invitrogen	A31571	IF	1:2000
Anti-rabbit Alexa 647	Donkey	Invitrogen	A31573	IF	1:2000
Anti-goat Alexa 647	Donkey	Invitrogen	A21447	IF	1:2000
Anti-rat Alexa 650	Donkey	ThermoFisher	SA5-10029	IF	1:200

Table S2. Quantitative RT-PCR primers.

Gene or transcript	Forward (5′–3′)	Reverse (5′–3′)
Actb	GGCTGTATTCCCCTCCATCG	CCAGTTGGTAACAATGCCATGT
Ppia	TGAGCACTGGAGAGAAAGGA	CCATTATGGCGTGTAAAGTCA
Gapdh	GCACAGTCAAGGCCGAGAAT	GCCTTCTCCATGGTGGTGAA
Lmna	CCTATCGAAAGCTGCTGGAG	CCTGAGACTGGGATGAGTGG
Lmnb1	CAACTGACCTCATCTGGAAGAAC	TGAAGACTGTGCTTCTCTGAGC
Lmnb2	GAGGACATTGCCTACAAGTTCAC	TTCCACACAAGGGTTGATG
Lamin C	GACAATGAGGATGACGACGAG	TTAATGAAAAGACTTTGGCATGG
Nup153	AAGCGAAGTGCTCCTTGTGAGG	TGGTAGTGGGTTGAAGAGCAGG
Nup98	CCTCTTGGTACAGGAGCCTTTG	TTGAGGTGCTGCTGGAGAACAG
Progerin	CTGAGTACAACCTGCGCTCA	AGTTCTGGGAGCTCTGGGCT
Snf7b	GCAGGACATTGCTGACCAGCAA	CAAGTTCCTCCAACTCTGCCATG
Sun1	AGCATCCTAAGCACTCGGT	TAGATGTCGGGCTGGATCAC
Sun2	CAGGATTGGAATGGTGGATTA	AGTGCCGTCTTGGTCTCATAA
Syne1	CTGCTGCTTATTGGACTCACCT	GAGGAGGACCGTTGGTATATCTG
Syne2	CAGGAGTATCCAGAACATTCCCG	AGACTGCTGTGCCTGGATTTCG
Tmpo	GGAGTGAATCCTGGTCCCATTG	GACTGTTGGAAGAGGAGTAGAGG
Vps2	AACCGAGCCATGAGAGAACTGG	ACGCACCAGGTCTTTTGCCATG
Vps20	GAGCAACTGCTCGATAGGACAG	CCCTCCATCACCTTCATCTCGA
Vps24	TCAGGTCAAGGAAAGCCGTGAG	TGCAGAGAACCAGCTACTCGCA
Vps4	GAGAACCAGAGTGAAGGCAAGG	TCCACCGTATGTTCGGCTTCTC
Vps60	GATGAGAGAGGGTCCTGCTAAG	CAGGTTGTCTCGCTGTTGCTCA

Table S3. Plasmid constructs.

Plasmid name	Protein	Constitutive or inducible	Drug selection
Prelamin A-pTRIPZ	Prelamin A	Inducible	Puromycin
Progerin-pTRIPZ	Progerin	Inducible	Puromycin
nf-Progerin-pTRIPZ	Non-farnesylated progerin	Inducible	Puromycin
Lmnb1-pTRIPZ	Lamin B1	Inducible	Puromycin
nf-*Lmnb1*-pTRIPZ	Non-farnesylated lamin B1	Inducible	Puromycin
LMNB2-pTRIPZ	Lamin B2	Inducible	Puromycin
Prelamin A-pCDH	Prelamin A	Constitutive	Blasticidin
Progerin-pCDH	Progerin	Constitutive	Blasticidin
NLS-CopGFP-pCDH	Nuclear localized GFP	Constitutive	Blasticidin
NLS-TurboRFP-pCDH	Nuclear localized RFP	Constitutive	Blasticidin
pLenti6-GFP-KASH2	GFP–KASH2	Constitutive	Blasticidin
pLenti6-GFP-KASH2ext	GFP–KASH2ext	Constitutive	Blasticidin

Full unedited gel for Figure 1A.

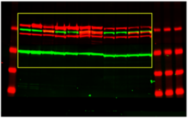

Antibodies

Lamin A/C
Lamin B1
Tubulin

Full unedited gel for Figure 3A.

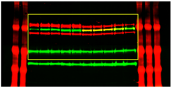

Antibodies

Lamin A/C
Lamin B1
Tubulin
Actin

Full unedited gel for Figure 3H.

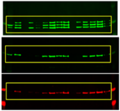

Antibodies

Lamin A/C
hLamin A/C
Lamin B1

Full unedited gel for Figure 4G.

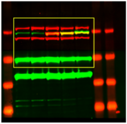

Antibodies

Lamin A/C
Lamin B1
Tubulin
Actin

Full unedited gel for Figure 5A.

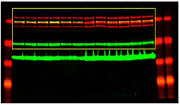

Antibodies

Lamin A/C
Lamin B1
Tubulin
Actin

Full unedited gel for Figure 5G.

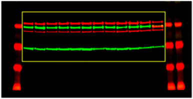

Antibodies

Lamin A/C
Lamin B1
Tubulin

Full unedited gel for Figure S1B.

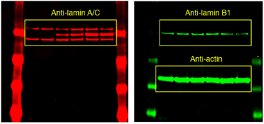

Full unedited gel for Figure S2A.

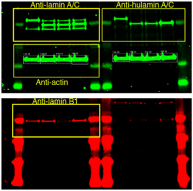

Full unedited gel for Figure S3A.

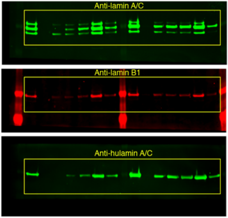

Full unedited gel for Figure S4A.

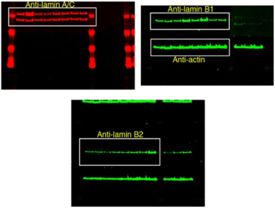

Full unedited gel for Figure S4B.

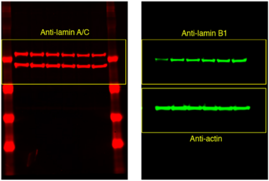

Full unedited gel for Figure S4F.

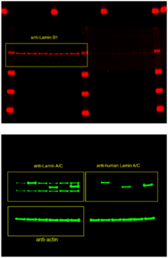

Full unedited gel for Figure S5D.

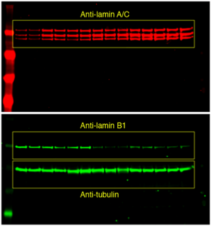

Supplement: Supplemental data [file jciinsight-6-151515-s186.pdf]
